# Characterization of monoamine oxidase-B (MAO-B) as a biomarker of reactive astrogliosis in Alzheimer’s disease and related dementias

**DOI:** 10.1007/s00401-024-02712-2

**Published:** 2024-04-03

**Authors:** Methasit Jaisa-aad, Clara Muñoz-Castro, Molly A. Healey, Bradley T. Hyman, Alberto Serrano-Pozo

**Affiliations:** 1 Department of Neurology, Massachusetts General Hospital, Boston, MA 02114 USA; 2 MassGeneral Institute for Neurodegenerative Disease, 114 16th St., Charlestown, MA 02129 USA; 3 Massachusetts Alzheimer’s Disease Research Center, Charlestown, MA 02129 USA; 4 Harvard Medical School, Boston, MA 02115 USA

**Keywords:** Alzheimer’s disease, Amyloid plaques, Astrocyte, Monoamine oxidase-B, Neurofibrillary tangles, Positron emission tomography

## Abstract

Reactive astrogliosis accompanies the two neuropathological hallmarks of Alzheimer’s disease (AD)—Aβ plaques and neurofibrillary tangles—and parallels neurodegeneration in AD and AD-related dementias (ADRD). Thus, there is growing interest in developing imaging and fluid biomarkers of reactive astrogliosis for AD/ADRD diagnosis and prognostication. Monoamine oxidase-B (MAO-B) is emerging as a target for PET imaging radiotracers of reactive astrogliosis. However, a thorough characterization of MAO-B expression in postmortem control and AD/ADRD brains is lacking. We sought to: (1) identify the primary cell type(s) expressing MAO-B in control and AD brains; (2) quantify MAO-B immunoreactivity in multiple brain regions of control and AD donors as a proxy for PET radiotracer uptake; (3) correlate MAO-B level with local AD neuropathological changes, reactive glia, and cortical atrophy; (4) determine whether the *MAOB* rs1799836 SNP genotype impacts MAO-B expression level; (5) compare MAO-B immunoreactivity across AD/ADRD, including Lewy body diseases (LBD) and frontotemporal lobar degenerations with tau (FTLD-Tau) and TDP-43 (FTLD-TDP). We found that MAO-B is mainly expressed by subpial and perivascular cortical astrocytes as well as by fibrous white matter astrocytes in control brains, whereas in AD brains, MAO-B is significantly upregulated by both cortical reactive astrocytes and white matter astrocytes across temporal, frontal, and occipital lobes. By contrast, MAO-B expression level was unchanged and lowest in cerebellum. Cortical MAO-B expression was independently associated with cortical atrophy and local measures of reactive astrocytes and microglia, and significantly increased in reactive astrocytes surrounding Thioflavin-S+ dense-core Aβ plaques. MAO-B expression was not affected by the *MAOB* rs1799836 SNP genotype. MAO-B expression was also significantly increased in the frontal cortex and white matter of donors with corticobasal degeneration, Pick’s disease, and FTLD-TDP, but not in LBD or progressive supranuclear palsy. These findings support ongoing efforts to develop MAO-B-based PET radiotracers to image reactive astrogliosis in AD/ADRD.

**Supplementary Information:**

The online version contains supplementary material available at 10.1007/s00401-024-02712-2.

## Introduction

The two main Alzheimer’s disease neuropathological changes (ADNC)—amyloid-β (Aβ) plaques and phospho-tau (pTau) neurofibrillary tangles—are closely surrounded and/or penetrated by reactive astrocytes and microglia [25, 44, 45], yet the role of these glial responses in the formation, maturation, and clearance of ADNC and in downstream neuronal and synaptic loss remains controversial [38]. Multimodal biomarker studies will be crucial to understand the relationship between ADNC and reactive glia [36, 37]. However, while cerebrospinal fluid (CSF), plasma, and PET imaging biomarkers of Aβ and pTau have been extensively cross-validated and correlated with postmortem neuropathological findings, the validation of biomarkers of reactive glia is lagging behind. In a previous quantitative neuropathological study, we found that the purported PET imaging biomarker of reactive microglia translocator protein 18 kDa (TSPO) is not only expressed by microglia but also by astrocytes, endothelial cells, and vascular smooth muscle cells, and that its expression level is not dramatically different between AD and control (CTRL) donors, nor does it correlate with the number of reactive microglia in AD [20]. Another more recent study has added to this controversy on TSPO [34].

Monoamine oxidase-B (MAO-B) has been recently proposed as a target for development of PET imaging radiotracers of AD-associated reactive astrogliosis [9]. However, the few MAO-B PET imaging studies published to date have reported conflicting results. Indeed, the initial studies using [^11^C]-Deuterium-l-Deprenyl ([^11^C]-DED)—an irreversible MAO-B inhibitor derived from the anti-parkinsonian drug selegiline—did not reveal clear differences between CTRL and sporadic AD individuals and pointed to an increased uptake in pre-symptomatic autosomal dominant AD mutation carriers with decreased uptake toward their expected age of onset [5, 41, 43]. More recent studies, however, indicate that the newer reversible MAO-B inhibitor (S)-(2-methylpyrid-5-yl)-6-[(3-18F-fluoro-2-hydroxy) propoxy]quinoline or ^18^F-SMBT-1 may discriminate CTRL from sporadic AD and individuals with other neurodegenerative diseases [23, 46–48].

MAO-B is an enzyme mainly localized at the mitochondrial outer membrane and that catalyzes the oxidation of monoamine neurotransmitters such as dopamine, tryptamine, tyramine, phenylethylamine, benzylamine, norepinephrine, and serotonin [53]. MAO-B is expressed in catecholaminergic neurons and astrocytes [15, 29], but the evidence for its upregulation in AD reactive astrocytes is scarce. Biochemical enzymatic assays [27] and phosphor-screen autoradiography studies [21, 27] have shown an increased MAO-B activity and a higher binding of MAO-B radiotracers, respectively, in AD versus CTRL brains, but cannot reveal the cellular source of MAO-B. Qualitative postmortem immunohistochemical studies have reported an increased MAO-B immunoreactivity in GFAP+ astrocytes around Aβ plaques [26, 49], however, quantitative neuropathological studies correlating MAO-B expression level with ADNC burden and measures of reactive gliosis are needed to better interpret PET imaging studies. Moreover, the *MAOB* *rs1799836* SNP has been reported to impact MAO-B enzymatic activity [3] but its effect on MAO-B expression by AD reactive astrocytes has not been investigated. Lastly, little is known about MAO-B brain expression levels in ADRD, including Lewy body diseases (LBD), primary tauopathies [e.g., Pick’s disease (PiD), progressive supranuclear palsy (PSP), and corticobasal degeneration (CBD)], and frontotemporal lobar degeneration with Tar DNA-binding protein of 43 kDa (FTLD-TDP).

We aimed to characterize MAO-B expression level in postmortem CTRL and AD brains. Specifically, we sought to: (1) identify the cell type(s) expressing MAO-B in CTRL and AD brains; (2) quantify MAO-B immunoreactivity in multiple regions from CTRL and AD brain donors as a proxy for PET radiotracer uptake; (3) correlate MAO-B expression level with the local burden of ADNC, reactive astrocytes and microglia, and cortical atrophy to inform multimodal biomarker studies; (4) determine whether the *MAOB* *rs1799836* SNP impacts brain MAO-B expression level; and (5) compare MAO-B expression levels in a disease-relevant region such as the dorsolateral prefrontal cortex across CTRL, AD, LBD, PiD, PSP, CBD, and FTLD-TDP brains.

## Materials and methods

### Human postmortem brain specimens

8-μm-thick formalin-fixed paraffin-embedded (FFPE) tissue sections from the temporal [including Brodmann area (BA) 38], frontal (BA8/9), and occipital cortex (BA17/18), and cerebellum of *n* = 52 donors spanning the AD neuropathological continuum from normal aging brain (CTRL, “not AD/low ADNC burden”) to severe AD (high ADNC burden) were obtained from the Massachusetts Alzheimer’s Disease Research Center (MADRC) Neuropathology Core Brain Bank. FFPE sections from the hippocampus of selected donors were also used for illustration purposes. Table 1 depicts the demographic (age, sex) and neuropathological (Thal amyloid phase, CERAD neuritic plaque score, and Braak neurofibrillary tangle stage) characteristics, and the *APOE* genotype of the *n* = 52 study donors split by ADNC burden [32]. Frozen samples from the cerebellum were also available for *n* = 50 donors for DNA purification and *MAOB* SNP genotyping. We validated the main findings of this study in a separate sample of *n* = 51 donors with intermediate or high ADNC burden, for which quantitative neuropathological data was available [44]. Specifically, we used Aβ+ area fraction, cortical thickness, and stereology-based count data of pTau+ (PHF1) neurofibrillary tangles, GFAP+ astrocytes, and CD68+ microglia from the temporal association neocortex of this validation sample, which were obtained following procedures detailed elsewhere [44].

**Table 1:** Demographic and neuropathological characteristics, and *APOE* genotype of the study donors along the AD continuum split by the burden of AD neuropathological changes

	AD neuropathological change (ADNC) burden			
	Not AD/low	Intermediate	High	*p *value
N	18	10	24	
Age at death (y)	86.0 ± 9.6	88.5 ± 10.2	79.6 ± 11.8	0.0092^a^
Sex, *n* female (%)	9 (50.0)	5 (50.0)	10 (41.7)	0.8348^b^
*APOE*ε4 carriers, *n* (%)	3 (5.8)	2 (3.9)	17 (32.7)	0.0003^b^
*APOE* genotype:				
*ε*2/*ε*3	3	2	0	0.0001^c^
*ε*3/*ε*3	12	6	6	
*ε*2/*ε*4	0	1	0	
*ε*3/*ε*4	3	0	15	
*ε*4/*ε*4	0	1	2	
Postmortem interval (h)	23.2 ± 10.1	28.2 ± 17.4	19.9 ± 11.4	0.2219^d^
Braak NFT stages:				
0	0	0	0	< 0.0001^c^
I/II	14	0	0	
III/IV	3	10	0	
V/VI	0	0	24	
Thal amyloid phases:				
0	5	0	0	< 0.0001^c^
1–2	6	0	0	
3	3	4	0	
4–5	1	6	24	
CERAD neuritic plaque scores:				
C0	8	0	0	< 0.0001^c^
C1	7	3	0	
C2	2	6	6	
C3	0	1	17	

^a^Kruskal–Wallis ANOVA

^b^Chi-square test

^c^Chi-square test for trend with Not AD/Low and Intermediate groups combined

^d^One-way ANOVA

In addition, 8-μm-thick FFPE sections from the frontal association cortex (BA8/9) of *n* = 70 donors with a primary neuropathological diagnosis of ADRD—comprising *n* = 30 LBD (including five with brainstem/limbic and 25 with neocortical Lewy body stage), *n* = 30 FTLD-Tau (including ten of each PiD, PSP, and CBD), and *n* = 10 FTLD-TDP—were also obtained from the MADRC Neuropathology Core Brain Bank (Table 2). Donors or their next-of-kin gave a written informed consent for the brain donation and this study was conducted under the MADRC Neuropathology Core Institutional Review Board.

**Table 2:** Demographic characteristics of the study donors with a primary neuropathological diagnosis of ADRD

	LBD^a^	PSP	CBD	PiD	FTLD-TDP	*p-*value^b^
*n*	30	10	10	10	10	
Age at death (y)	77.2 ± 9.3	73.4 ± 5.5	69.5 ± 9.1	67.2 ± 11.4	69.2 ± 10.1	< 0.0001^c^
Sex, *n* female (%)	5 (16.7)	5 (50.0)	5 (50.0)	5 (50.0)	5 (50.0)	0.2092^d^

^a^The LBD group includes *n* = 5 donors with brainstem/limbic Lewy body stage and *n* = 25 with neocortical Lewy body stage

^b^Contrasts included “Not AD/Low,” “Intermediate,” and “High ADNC burden” groups from Table 1

^c^Kruskal–Wallis ANOVA with Dunn’s multiple comparison post-test (PSP vs. Intermediate ADNC: *p* = 0.0420; CBD vs. Not AD/Low ADNC: *p* = 0.0028; CBD vs. Intermediate ADNC: *p* = 0.0028; PiD vs. Not AD/Low ADNC: *p* = 0.0009; PiD vs. Intermediate ADNC: *p* = 0.0010; FTLD-TDP vs. Not AD/Low ADNC: *p* = 0.0040; FTLD-TDP vs. Intermediate ADNC: *p* = 0.0038)

^d^Chi-square test. Abbreviations: LBD = Lewy body diseases; PSP = progressive supranuclear palsy; CBD = corticobasal degeneration; PiD = Pick’s disease; FTLD-TDP = frontotemporal lobar degeneration—Tar DNA-binding protein of 43 kDa

### Multiplex fluorescent immunohistochemistry

To determine the cell type expressing MAO-B, we performed double fluorescent immunohistochemistry with antibodies for MAO-B and cell-type-specific markers on FFPE sections from the temporal association cortex of selected CTRL and AD donors followed by laser confocal microscopy. Briefly, FFPE sections were dewaxed in xylenes and rehydrated in decreasing concentrations of ethanol. Next, sections were microwaved in boiling citrate buffer (0.01 M, pH 6.0 with 0.05% Tween20) for 20 min at 95 °C for antigen retrieval. Subsequently, sections were blocked with 10% normal donkey serum (NDS) in Tris-buffered saline (TBS) for 1 h at room temperature (RT) prior to incubation with primary antibodies in 5%NDS/TBS at 4 °C overnight. On the following day, sections were washed with TBS before incubation with secondary antibodies at 1:200 in 5%NDS/TBS for 2 h at RT, washed with TBS, and counterstained with Thioflavin-S (ThioS, Sigma, T1892) by immersion in 0.05% ThioS in 50% ethanol for 8 min, followed by differentiation with 80% ethanol for 10 s. Finally, sections were incubated with Autofluorescence Eliminator Reagent (Millipore, 2160) for 5 min at RT to quench endogenous tissue autofluorescence, cleared with serial 70% ethanol washes of 20 s each, rehydrated in TBS, and coverslipped with Fluoromount-G mounting media with DAPI (Southern Biotech, 0100-01). The following primary antibodies were used: rabbit anti-MAO-B monoclonal antibody (raised against amino acids 1-100 of human MAO-B, clone EPR7102, Abcam, ab133270, 1:500), mouse anti-Aβ monoclonal antibody (clone 6E10, BioLegend, 803003, 1:100), mouse anti-ALDH1L1 monoclonal antibody (clone N103/39, Millipore, MABN495, 1:500), mouse anti-CD31 monoclonal antibody (clone 89C2, Cell Signaling Technology, #3528, 1:100), mouse anti-GFAP monoclonal antibody (clone G-A-5, Sigma, G3893, 1:1000), goat anti-IBA1 polyclonal antibody (Abcam, ab107159, 1:100), mouse anti-microtubule-associated protein 2 (MAP2) monoclonal antibody (clone SMI-52, Biolegend, 801801, 1:250), mouse anti-myelin basic protein (MBP) monoclonal antibody (clone 1, Millipore, MAB382, 1:500), mouse anti-platelet derived growth factor receptor-β (PDGFRβ) monoclonal antibody (clone D-6, Santa Cruz Biotechnology, sc-374573, 1:200). Secondary antibodies included a donkey anti-rabbit Cy3-conjugated antibody (Jackson ImmunoResearch) for MAO-B and species-appropriate donkey antibodies conjugated with Cy5 (ThermoFisher Scientific) for all other primary antibodies.

### Peroxidase-DAB immunohistochemistry

For quantitative analyses, we immunostained FFPE sections from all donors with the peroxidase-3,3′-diaminobenzidine (DAB) method using the BOND Polymer Refine Detection kit (Leica Biosystems, DS9800) in a Leica BOND RX Fully Automated Research Stainer (Leica Biosystems). Specifically, the following primary antibodies were used: rabbit anti-MAO-B monoclonal antibody (clone EPR7102, Abcam, ab133270, 1:1000), rabbit anti-Aβ monoclonal antibody (clone D52D4, Cell Signaling Technology, 1:2000), mouse anti-CD68 monoclonal antibody (clone KP-1, Dako, M0814, 1:500), mouse anti-GFAP monoclonal antibody (clone G-A-5, Sigma-Aldrich, G3893, 1:20,000), and mouse anti-pTau^Ser396/404^ monoclonal antibody (clone PHF1, kind gift from Dr. Peter Davies, 1:500). MAO-B immunohistochemistry was performed in all four regions (temporal, frontal, occipital, and cerebellum) of all donors in Table 1 except for one donor for which cerebellum was not available, in the hippocampus of selected donors in Table 1, as well as in frontal sections from all donors in Table 2. Aβ, CD68, GFAP, and pTau immunostains were only performed in temporal cortex sections from all donors in Table 1. The automated immunohistochemistry protocol consists of: (1) baking at 80 °C for 60 min; (2) dewaxing using BOND Dewax solution (Leica Biosystems) followed by rehydration; (3) heat-induced epitope retrieval (HIER) with citrate (ER1) at 100℃ for either 20 min (MAO-B, Aβ, and pTau) or 30 min (CD68) except for GFAP, which did not require this step; (4) blocking of endogenous peroxidase activity with a 4% (v/v) hydrogen peroxide solution in methanol for 20 min; (5) primary antibody incubation at 37℃ for 35 min except for anti-CD68 antibody, which was incubated for 60 min; (6) secondary antibody incubation with post-primary solution containing 10 g/mL Poly-HRP IgG in 0.01% 2-methylisothiazol-3(2*H*) for 10 min followed by polymer solution containing 25 g/mL Poly-HRP IgG in 0.01% 2-methylisothiazol-3(2*H*) for 8 min; (7) DAB substrate-peroxidase reaction for 1 min; (8) counterstaining with 0.1% hematoxylin for 15 min; and (9) washes with wash buffer and double-distilled water. Immunostained sections were dehydrated in increasing concentrations of ethanol and coverslipped with Permount mounting media (Fisher Scientific, SP15-500).

### Microscopy

#### Laser confocal microscopy

To study the expression of MAO-B in various brain cell types, selected sections subjected to multiplex fluorescent immunohistochemistry for MAO-B and cell-type-specific markers were imaged in an Olympus FV3000 confocal laser scanning inverted microscope (Olympus, Tokyo, Japan), which is equipped with 405, 488, 561, and 640 nm lasers as well as two regular and two high-sensitivity spectral detectors. Briefly, we used a 40 × /0.95 air objective and imaged *z*-stacks with the sequential mode of acquisition and different detectors for MAO-B and the cell-type-specific marker of interest to minimize the risk of bleeding through, and then obtained maximum intensity projections of the *z*-stacks.

#### Whole-slide bright-field microscopy

Sections immunostained for MAO-B, Aβ, CD68, GFAP, and pTau with the peroxidase-DAB method were scanned in a VS120 Olympus Virtual Slide Microscope to obtain whole-slide images under a 40 × /0.95 air objective.

### Quantitative neuropathological methods

#### Measurement of immunoreactive area fractions

We used the open access software QuPath (version 0.3.2) for all quantitative neuropathological analyses. To account for the variable distribution of the signal across the entire section, we outlined the cortex (and for MAO-B also the white matter) of the whole temporal, frontal, and occipital sections, and converted those outlines into annotations. For cerebellar sections, we only annotated the cortex and white matter of a single folium because one was representative of the entire section. We chose the QuPath pixel classifier tool because this outperformed the thresholder tool in our pilot studies. Specifically, the pixel classifier parameters were set at full resolution (0.17 μm/pixel), default multiscale features (scale 1.0, Gaussian filter, no local normalization), and smoothing sigma 0.25 for all markers. A separate pixel classifier was trained for each marker quantification by providing the artificial neural network (ANN-MLP) with 25 positive annotations (i.e., DAB-positive pixels) and 25 negative annotations (i.e., background and hematoxylin-positive pixels) of the temporal, frontal, and occipital sections of five CTRL and five AD donors (total training sample per classifier: 1500, 750 positive and 750 negative annotations). Next, we processed the images in project batches using automated scripts for each pixel classifier. The area fraction was defined as the % area occupied by DAB-positive pixels with respect to the area of the entire cortical or white matter annotation.

#### Measurement of cortical thickness

To obtain the temporal lobe cortical thickness, we applied the QuPath line annotation tool to GFAP-immunostained whole-section images and measured the shortest distance between the pial surface and the white matter (i.e., line perpendicular to both) in 20 random sites distributed throughout the cortical ribbon. The cortical thickness was defined as the average of those 20 measurements, as described before [44]. The GFAP-immunostained sections were chosen for cortical thickness measurements because the high GFAP expression by the glia limitans serves as fiduciary landmark of the cortical surface and the higher GFAP expression in white matter versus cortex facilitates the gray-white matter differentiation.

#### Measurement of plaque-centered area fractions

To determine whether the expression of MAO-B is spatially associated with dense-core Aβ plaques, we performed plaque-centered quantitative analyses in FFPE sections from the temporal cortex of *n* = 10 high ADNC (AD) donors and *n* = 10 not AD/low ADNC (CTRL) donors fluorescently immunostained for MAO-B and GFAP (as positive control) and counsterstained with ThioS. Briefly, in each AD donor, *n* = 50 ThioS+ dense-core Aβ plaques were randomly selected and outlined in the green channel of the whole-slide image with the appropriate tool in QuPath ensuring that all layers of the cortical ribbon were equally represented, while being blind-folded to the expression level of MAO-B in the TRITC channel and of GFAP in the Cy5 channel. Next, a 50 μm concentric halo was added to each selected plaque and *n* = 50 regions-of-interest of similar size but far (> 50 μm) from any ThioS+ plaque were overlaid on the whole-slide image with the appropriate tools in QuPath software. For each CTRL donor, *n* = 50 regions-of-interest of similar size to the AD plaques were added onto the cortical ribbon as sham plaques, together with their 50-μm halo, and *n* = 50 more regions-of-interest far (> 50 μm) from those sham plaques. The MAO-B+ and GFAP+ area fractions were measured in the three types of regions-of-interest, i.e., ThioS+ or sham plaques, peri-plaque halo (≤ 50 μm), and areas distant (> 50 μm) from ThioS+ or sham plaques.

### *MAOBrs1799836* SNP genotyping

To genotype the *MAOB* *rs1799836* SNP, we used a commercially available Taqman PCR assay on genomic DNA extracted from frozen cerebellar samples. Briefly, we purified genomic DNA from ≈ 25 mg of frozen cerebellum using the PureLink Genomic DNA Extraction Mini Kit (ThermoFisher Scientific, K182002) following manufacturer’s instructions. We measured the DNA concentration in a DS-11 spectrophotometer (DeNovix Inc) and prepared 1.8 ng/μL working dilutions for the Taqman PCR assay. The latter reaction volume was 25 μL comprising 1.25 μL of 20 × TaqMan *MAOB* *rs1799836* genotyping assay (ThermoFisher Scientific, Assay ID C—8878790_10), 12.50 μL of 2 × TaqMan Fast Universal PCR Master Mix, no AmpErase UNG (Thermo Scientific, 4352042), and 11.25 μL of DNA sample (20 ng). DNA samples were run in duplicates in 96-well plates (Bio-Rad) in a Bio-Rad CFX96 Touch Real-Time PCR Detection System with the following protocol: 95 °C × 10 min (ramp 1 °C/s), 95 °C × 15 s, and 60 °C × 1 min, for 45 cycles. Principal component analysis of VIC versus FAM fluorescence [corresponding to base A (major allele) vs. G (minor allele), respectively] enabled allele discrimination and genotype assignment (AA, AG, or GG).

### SDS-PAGE and western blot

To validate the specificity of the anti-MAO-B antibody used in the immunohistochemical studies, we performed western blot in *MAOB*-overexpressing and knock-down human cell line lysates and human recombinant MAO-A and MAO-B proteins. The human recombinant MAO-A protein was purchased from LS-Bio (LS-G3233), whereas the human recombinant MAO-B protein was purchased from Abcam (ab82944), both purified from *Escherichia coli* with a 6xHis-tag. The human cell line lysates were kindly provided by Dr. Boyang (Jason) Wu (Washington State University, Spokane, WA) and consisted of lysates from PrSC human prostate stroma cells stably expressing either a control plasmid or a *MAOB* plasmid, and lysates from HepG2 human liver carcinoma cells stably expressing either a control shRNA or one of two anti-*MAOB* shRNAs [50]. Briefly, sample protein concentration was measured with the Pierce BCA Protein Assay Kit (ThermoFisher Scientific, 23225). After boiling at 95 °C for 5 min with 10X sample reducing agent (NuPAGE, ThermoFisher Scientific, NP0009) and 4X protein sample loading buffer (LI-COR Biosciences, P/N: 928-40004) for protein denaturalization, samples (4 μg for recombinant proteins, 10 μg for cell lysates) were loaded onto a 4–12% Bis–Tris gradient gel (NuPAGE, ThermoFisher Scientific, NP0343BOX) together with Precision Plus Dual Color Protein Standards (Bio-Rad, 1610374). SDS-PAGE was run in MOPS SDS running buffer (NuPAGE, ThermoFisher Scientific, NP0001) at 130 V for ~ 2 h. The gel proteins were transferred to a nitrocellulose membrane using a wet method at 310 mA for 2 h in ice. The membrane was blocked with Intercept Blocking Buffer (TBS) (LI-COR Biosciences, P/N 927-50000) for 1 h at RT, followed by incubation with primary antibodies in the Intercept Blocking Buffer (TBS) overnight at 4 °C. We used a mouse monoclonal anti-β-actin clone AC-15 (Sigma-Aldrich, A5541, 1:3000) as housekeeping loading control. Next day, the membrane was washed with TBS-Tween20 and incubated with the appropriate near-infrared fluorescent secondary antibodies (LI-COR Biosciences, 1:5000) for 1 h at RT. Finally, the membrane was thoroughly washed with TBS-Tween20 followed by TBS and scanned in an Odyssey CLx Imager (LI-COR Biosciences).

### Statistical analyses

We classified donors in three groups corresponding to their ADNC burden [32]. Normality of all datasets was determined by D’Agostino-Pearson Omnibus test. We ran one-way ANOVA with Tukey’s multiple comparison test if all datasets were normally distributed and Kruskal–Wallis ANOVA with Dunn’s multiple comparison test if one or more datasets were non-normally distributed. For correlation analyses between MAO-B area fraction and other neuropathological measures, we used Spearman’s rank correlation tests (since MAO-B area fraction was non-normally distributed) and simple linear regression. To investigate the effects of sex and *MAOB* *rs1799836* SNP genotype on MAO-B area fraction, we used two-way ANOVA with ADNC burden and either sex or *MAOB* *rs1799836* SNP genotype as factors and the sex × ADNC burden or genotype × ADNC burden interaction terms. To examine the independent associations between MAO-B expression and all other neuropathological measures, we built multiple linear regression models with temporal cortex MAO-B area fraction as outcome variable; temporal cortex Aβ, pTau, GFAP, and CD68 measures, and cortical thickness as independent variables; and age at death as co-variate (to account for possible influences of aging on cortical thickness, GFAP immunoreactivity, and diffuse Aβ deposits). Lastly, for the plaque-centered analyses, we used mixed effects models with MAO-B or GFAP area fraction as outcome variable, diagnosis (CTRL vs. AD) and location (sham or ThioS+ plaque, ≤ 50 μm peri-plaque halo, or > 50 μm), with or without a diagnosis × location interaction term, as fixed effects, and controlling for donor ID (random effect). The significance level was set at a two-sided *p* < 0.05 in all statistical analyses. All statistical analyses and graphs were performed with GraphPad Prism version 9.4.1 (GraphPad Inc., La Jolla, CA) except mixed-effect models, which were run in STATA version 15.0 (StataCorp, LLC., College Station, TX).

## Results

### Quantitative neuropathological characterization of control and AD brain donors

**Fig. 1:**
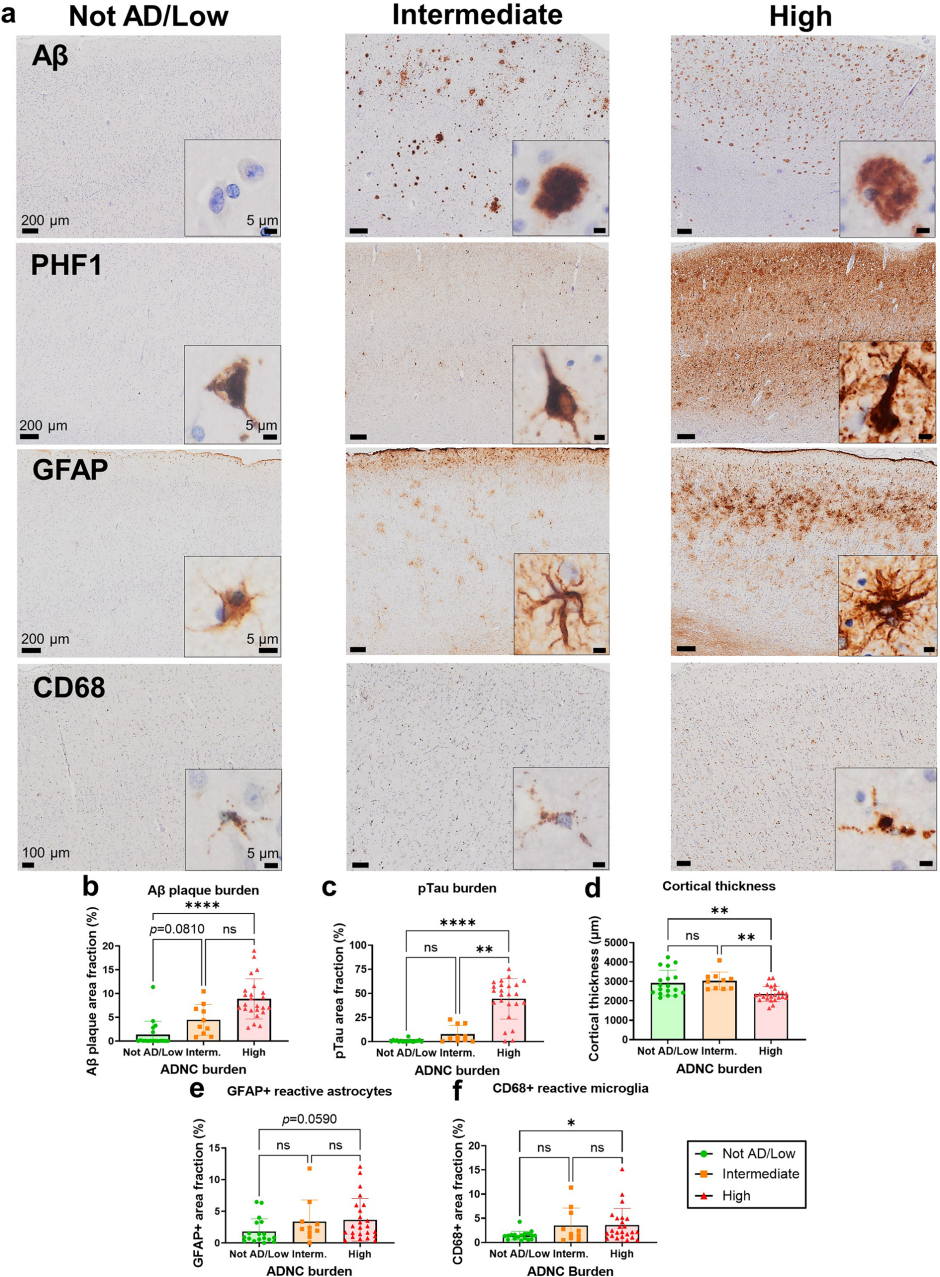
Quantitative neuropathological characterization of study donors. **a** Photomicrographs of Aβ, pTau (PHF1), GFAP, and CD68 immunohistochemistry in the temporal association neocortex of representative study donors with different ADNC burdens. Scale bars: 200 μm except CD68 100 μm, insets 5 μm. **b**–**f** Quantitative neuropathological measures of **b** Aβ+ area fraction, **c** pTau+ area fraction, **d** cortical thickness, **e** GFAP+ area fraction (reactive astrocytes), and **f** CD68+ area fraction (reactive microglia) in the temporal association neocortex of study donors split by ADNC group (ns = non-significant, *p < 0.05, **p < 0.01, ****p < 0.0001, one-way ANOVA with Tukey’s post-test or Kruskal–Wallis ANOVA with Dunn's post-test)

We first characterized the ADNC and reactive gliosis of CTRL and AD donors via quantitative immunohistochemistry for Aβ, pTau (PHF1, pTau^Ser396/404^), reactive astrocytes (GFAP), and reactive microglia (CD68), and also measured the cortical thickness in the temporal association neocortex. Figure 1 shows representative images of these immunostainings and the results of these neuropathological quantitative analyses by ADNC burden. As expected, high ADNC burden donors exhibited significantly higher levels of Aβ plaques and pTau relative to CTRL (i.e., not AD/low ADNC burden), with intermediate ADNC burden donors displaying intermediate levels between CTRL and definite AD donors. High ADNC burden donors had significantly more severe atrophy (i.e., lower cortical thickness) than intermediate ADNC burden and not AD/low ADNC burden donors, who did not differ in cortical thickness. Lastly, we found a marginally significant (*p* = 0.0590) increase in GFAP+ area fraction and a significant increase in CD68+ area fraction in high ADNC burden versus not AD/low ADNC burden donors, with intermediate ADNC burden donors exhibiting intermediate levels of reactive gliosis. Thus, we concluded that this sample is representative of the normal aging-severe AD neuropathological continuum.

### MAO-B is mainly expressed by perivascular astrocytes in the normal brain, and upregulated by reactive astrocytes around Aβ plaques in the AD cortex

To validate the specificity of the MAO-B antibody used for immunohistochemistry, we performed western blot on lysates from human cell lines stably overexpressing or downregulating *MAOB* as well as on recombinant MAO-B and MAO-A proteins. Western blot revealed a single band of ~ 59 kDa which increased upon *MAOB* overexpression and virtually disappeared upon *MAOB* silencing with specific shRNAs in human cell lines. Moreover, recombinant MAO-B produced a higher band (~ 62 kDa) due to its His-tag, whereas no band was detectable with recombinant MAO-A, thus confirming the specificity of the antibody for MAO-B (Supplemental Fig. S1).

**Fig. 2:**
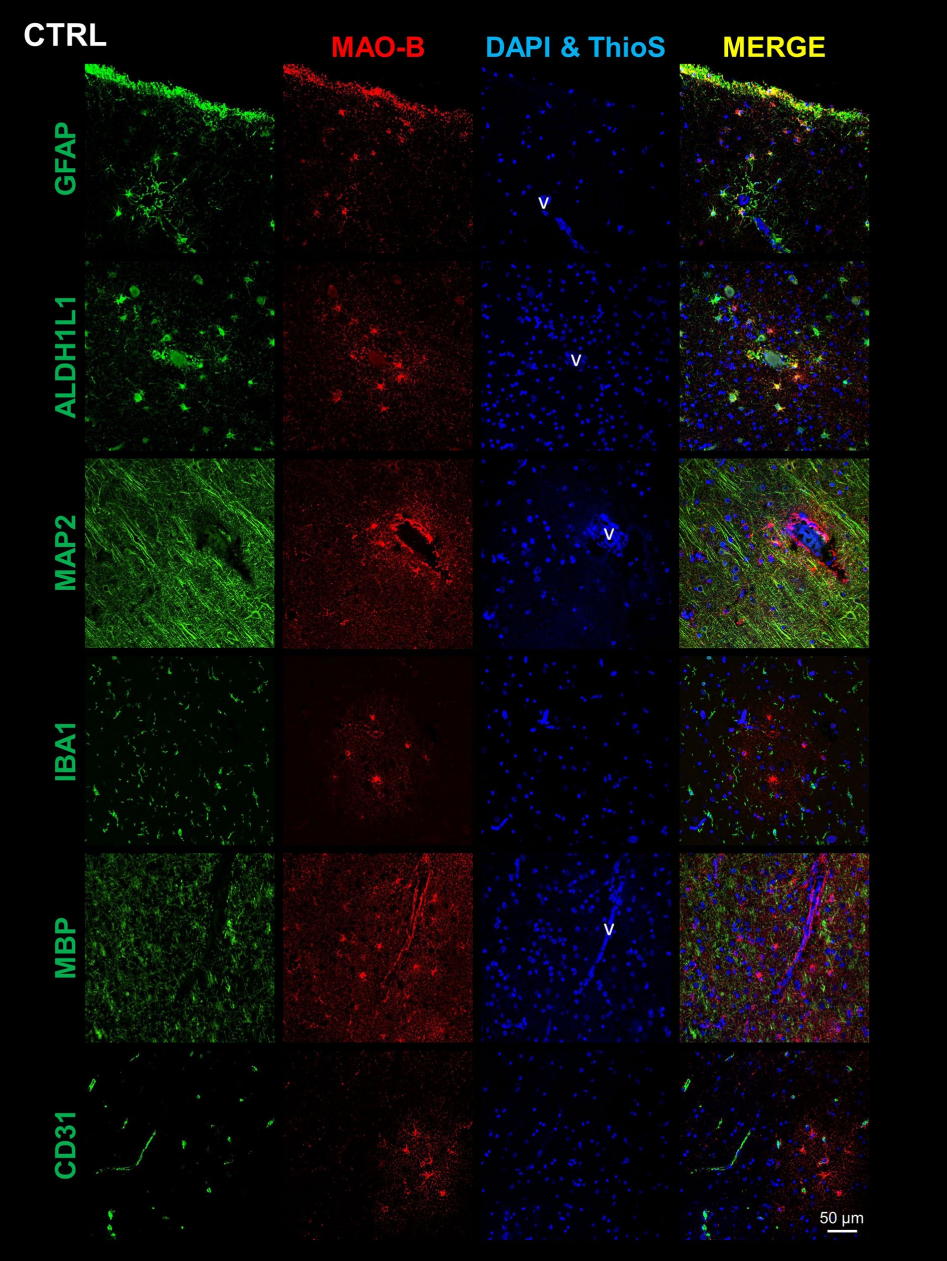
Astrocytes are the main cell type expressing MAO-B in the normal cortex. Double fluorescent immunohistochemistry for MAO-B and cell type-specific markers of either astrocytes (GFAP and ALDH1L1), neurons (MAP2), microglia (IBA1), oligodendrocytes (MBP), or endothelial cells (CD31) in the temporal neocortex of representative control (CTRL) donors. Confocal microscopy images show that MAO-B co-localizes mainly with subpial and perivascular astrocytes in the CTRL cortex, but not with the other cell types. Images were taken from cortical layers II–III for all markers except for GFAP, which also includes layer I subpial astrocytes, and MBP, which was taken from layers V–VI. Scale bar: 50 μm

**Fig. 3:**
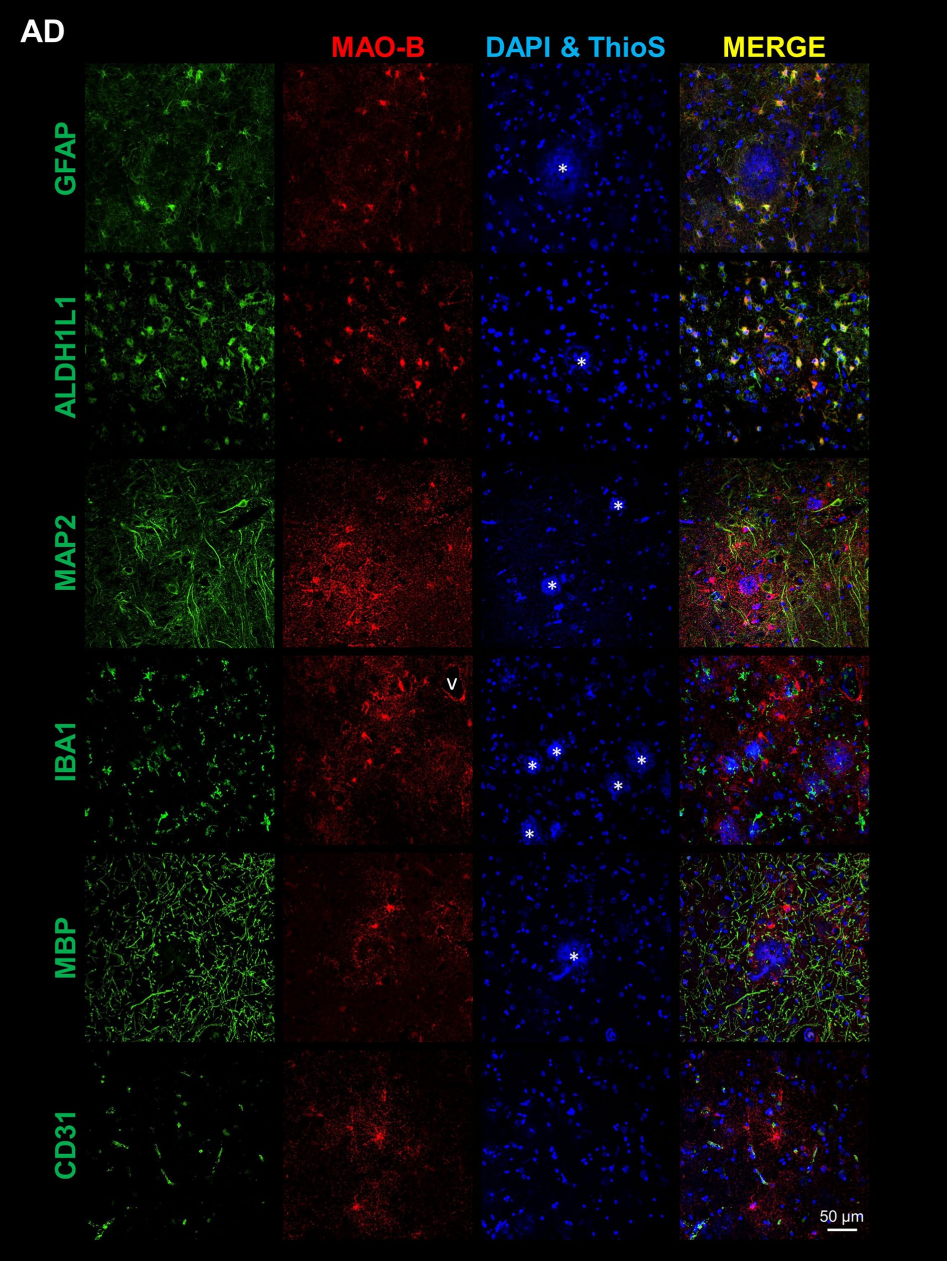
Astrocytes are the main cell type expressing MAO-B in the AD cortex. Double fluorescent immunohistochemistry for MAO-B and cell type-specific markers of either astrocytes (GFAP and ALDH1L1), neurons (MAP2), microglia (IBA1), oligodendrocytes (MBP), or endothelial cells (CD31) in the temporal neocortex of representative AD donors. Confocal microscopy images reveal that MAO-B co-localizes with reactive astrocytes around Aβ plaques in the AD cortex but has little or no colocalization with the other cell types. Images were taken from cortical layers II–III for all markers except for MBP, which was taken from layers V–VI. Scale bar: 50 μm

Next, to determine the cell type(s) expressing MAO-B, we performed double fluorescence immunohistochemistry and confocal microscopy for MAO-B and various cell type-specific markers on FFPE sections from the temporal association cortex. Co-immunostaining of MAO-B with GFAP and ALDH1L1 revealed colocalization of MAO-B with astrocytes in both CTRL (Fig. 2 and Supplemental Fig. S2) and AD donors (Fig. 3 and Supplemental Fig. S3). In CTRL brains, MAO-B was predominantly expressed by both subpial and perivascular astrocytes in the cortex (Fig. 2) as well as by fibrous astrocytes, particularly those surrounding blood vessels, in the white matter (Supplemental Fig. S2). In the AD cortex, MAO-B was highly expressed by many GFAP+ reactive astrocytes surrounding Aβ plaques (Fig. 3), whereas in the AD white matter it was apparent in both perivascular and non-perivascular astrocytes (Supplemental Fig. S3). In hippocampal and cerebellar sections immunostained with the peroxidase-DAB method we had noted that only rare pyramidal neurons were immunoreactive for MAO-B in the hippocampal CA1 region from either CTRL or AD donors, whereas many granular cells in the dentate gyrus did express MAO-B (Supplemental Fig. S4a) and cerebellar Purkinje cells exhibited very limited or no immunostaining in both CTRL and AD donors (Supplemental Fig. S4b). Confocal microscopy confirmed very low MAO-B expression, when detectable, in cortical pyramidal MAP2+ neurons, and no expression at all in IBA1+ microglia, MBP+ oligodendrocytes, or CD31+ endothelial cells in either cortex (Figs. 2 and 3 and Supplemental Fig. S5) or white matter (Supplemental Figs. S2 and S3). Similarly, cortical PDGFRβ+ pericytes (Supplemental Fig. S6) did not appear to express MAO-B. Negative control images of sections immunostained only for MAO-B ruled out “bleeding through” as the explanation for the colocalization with astrocyte markers (not shown).

### MAO-B expression is increased in AD versus control neocortex and white matter but not in cerebellum

**Fig. 4:**
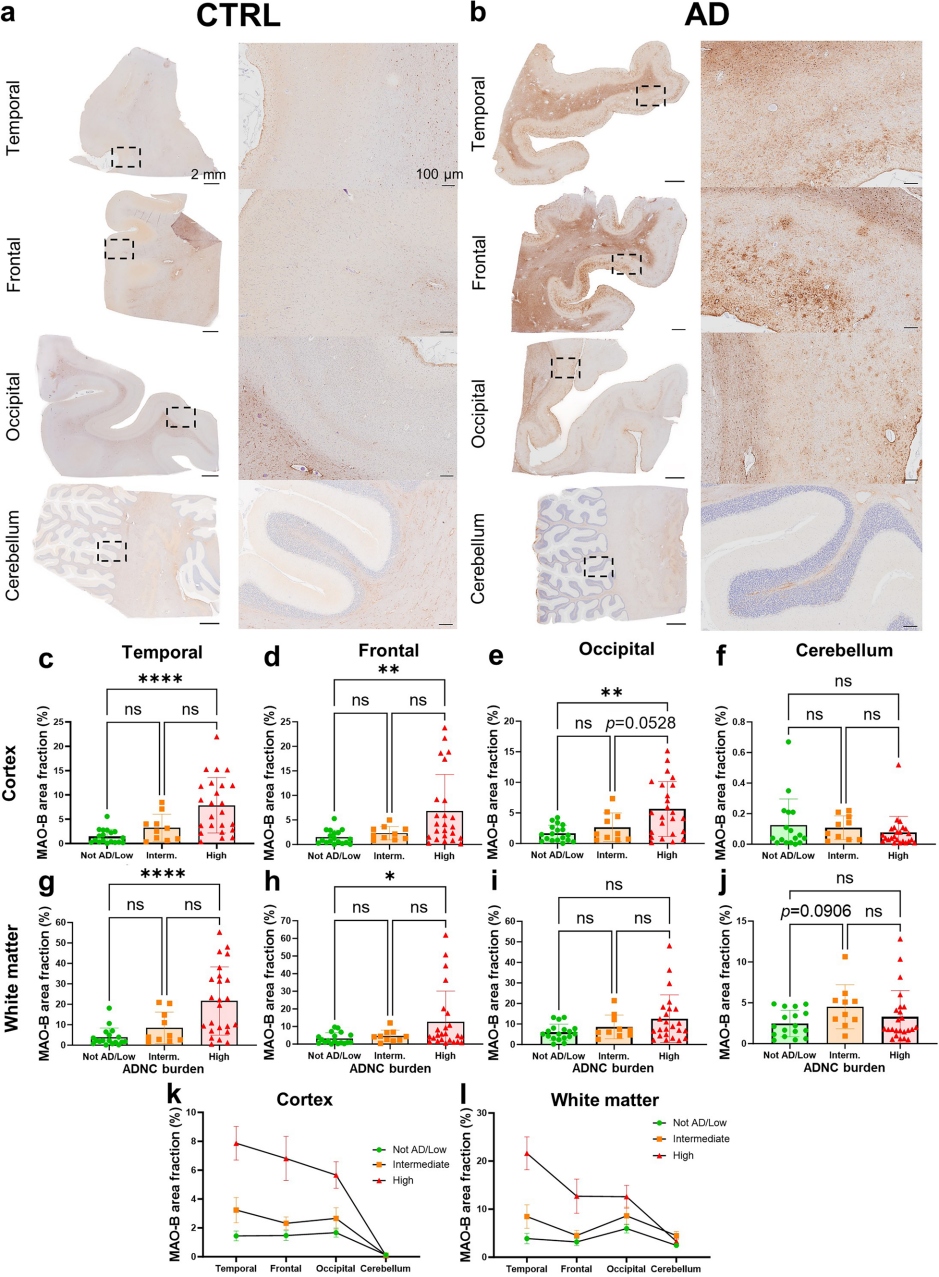
MAO-B is upregulated in AD versus CTRL brains. **a**, **b** Representative whole-slide images of MAO-B immunohistochemistry with the peroxidase-DAB method and higher power inset of **a** control (CTRL) and **b** AD donors across multiple brain regions. Scale bars: whole-slide images 2 mm, insets 200 µm. Comparative analysis of MAO-B expression across brain regions and ADNC burden groups revealed that MAO-B-immunoreactive area fraction is increased in AD (High ADNC) versus CTRL (Not AD/Low ADNC) donors in both **c**–**e** cortex and **g**–**i** white matter of temporal, frontal, and occipital lobes, but not in **f**, **j** cerebellum. **k**, **l** Moreover, trend plots across all regions analyzed demonstrated that the cerebellum has the lowest MAO-B expression level, especially the cerebellar cortex (ns = non-significant; *p < 0.05, **p < 0.01, ****p < 0.0001, Kruskal–Wallis ANOVA with Dunn’s post-test)

Once established that MAO-B is mainly expressed by cortical and white matter astrocytes, we sought to quantify MAO-B expression via automated immunohistochemistry with the peroxidase-DAB method, and measure MAO-B+ area fraction in both cortex and white matter of temporal, frontal, occipital, and cerebellar regions. We found significantly higher MAO-B expression in AD (high ADNC burden) than in CTRL (not AD/low ADNC burden) in temporal cortex and white matter, frontal cortex and white matter, and occipital cortex, whereas MAO-B+ area fraction did not significantly differ across ADNC groups in occipital white matter, cerebellar cortex, and cerebellar white matter (Fig. 4). Of note, the cerebellar cortex displayed the lowest MAO-B expression of all regions-of-interest in all donors, very close to zero. Also of note, MAO-B+ area fraction did not differ by sex (Supplemental Fig. S7) and did not significantly correlate with postmortem interval (Supplemental Fig. S8) or age at death (data not shown).

### MAO-B expression correlates with quantitative neuropathological measures of AD neuropathological changes, cortical atrophy, and reactive gliosis

**Fig. 5:**
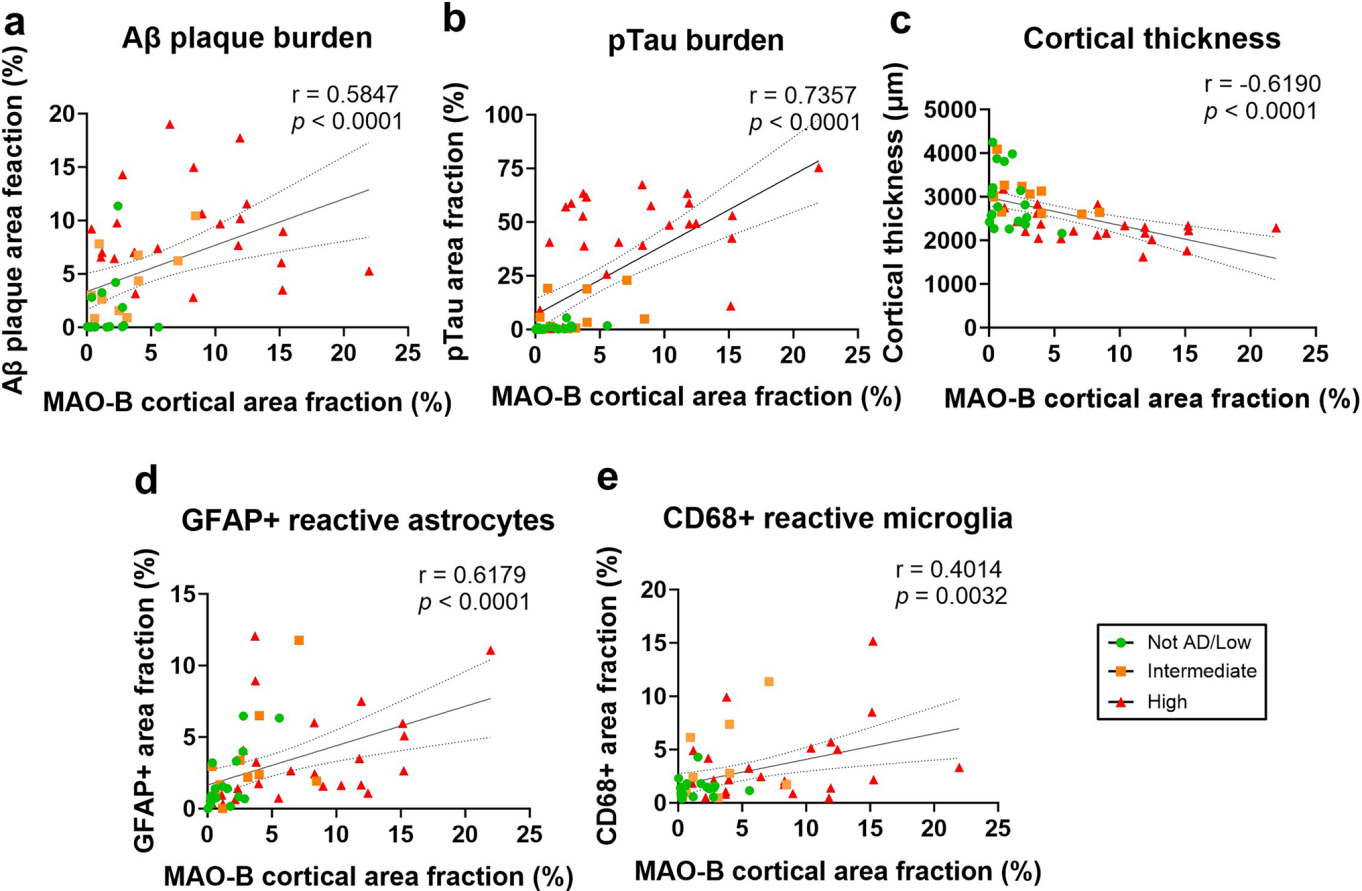
MAO-B expression correlates with local AD neuropathological measures, glial responses, and cortical atrophy. Correlation plots of MAO-B+ area fraction and **a** Aβ+ area fraction, **b** pTau+ area fraction, **c** cortical thickness, **d** GFAP+ area fraction (reactive astrocytes), and **e** CD68+ area fraction (reactive microglia) obtained in the temporal neocortex of study donors (*n* = 52). Spearman’s rank correlation tests (correlation coefficients and *p* values) are shown in each figure

Next, to determine the possible drivers of MAO-B expression level, we investigated correlations between MAO-B+ area fraction and ADNC burden (Aβ+ and pTau+ area fractions), cortical thickness, and reactive gliosis (GFAP+ and CD68+ area fractions for reactive astrocytes and microglia, respectively) in the temporal neocortex. Univariate analyses (Spearman’s rank test) revealed statistically significant positive correlations between MAO-B+ area fraction and Aβ +, pTau +, GFAP +, and CD68+ area fractions as well as a statistically significant negative correlation between MAO-B+ area fraction and cortical thickness (Fig. 5 and Table 3). A multivariable linear regression analysis with MAO-B+ area fraction as dependent variable; Aβ +, pTau +, GFAP +, and CD68+ area fractions, and cortical thickness as independent variables, and adjusted by age at death confirmed that MAO-B expression level is independently associated with pTau+ and CD68+ area fractions and showed trends toward a positive independent association with GFAP+ area fraction (*p* = 0.0997) and a negative association with cortical thickness (*p* = 0.0627), but no significant association with Aβ+ area fraction (Table 4).

**Table 3:** Univariate correlations of quantitative neuropathological measures obtained in the temporal association neocortex of the study donors along the AD continuum

	MAO-B+ area fraction	Aβ+ area fraction	pTau+ area fraction	Cortical thickness	GFAP+ area fraction	CD68+ area fraction
MAO-B+ area fraction	1.0					
Aβ+ area fraction	0.5847****	1.0				
pTau+ area fraction	0.7357****	0.7212****	1.0			
Cortical thickness	− 0.6190****	− 0.3402*	− 0.5036***	1.0		
GFAP+ area fraction	0.6179****	0.2565^ns^	0.5215****	− 0.3974**	1.0	
CD68+ area fraction	0.4014**	0.2930*	0.3092*	− 0.3154*	0.1202^ns^	1.0

Spearman’s rank test

*ns *non-significant

*p < 0.05; **p < 0.01; ***p < 0.001; ****p < 0.0001

**Table 4:** Multivariable linear regression analysis of MAO-B expression in the temporal association neocortex of the study donors along the AD continuum

Variables	Estimate	95% CI	*p* value
Age	− 0.0006586	− 0.1082 to 0.1069	0.9902
Aβ+ area fraction	0.1093	− 0.1640 to 0.3826	0.4244
pTau+ area fraction	0.06961	0.002458 to 0.1368	0.0425
Cortical thickness	− 0.002209	− 0.004541 to 0.0001227	0.0627
GFAP+ area fraction	0.3194	− 0.06342 to 0.7023	0.0997
CD68+ area fraction	0.3645	0.009851 to 0.7191	0.0442

**Fig. 6:**
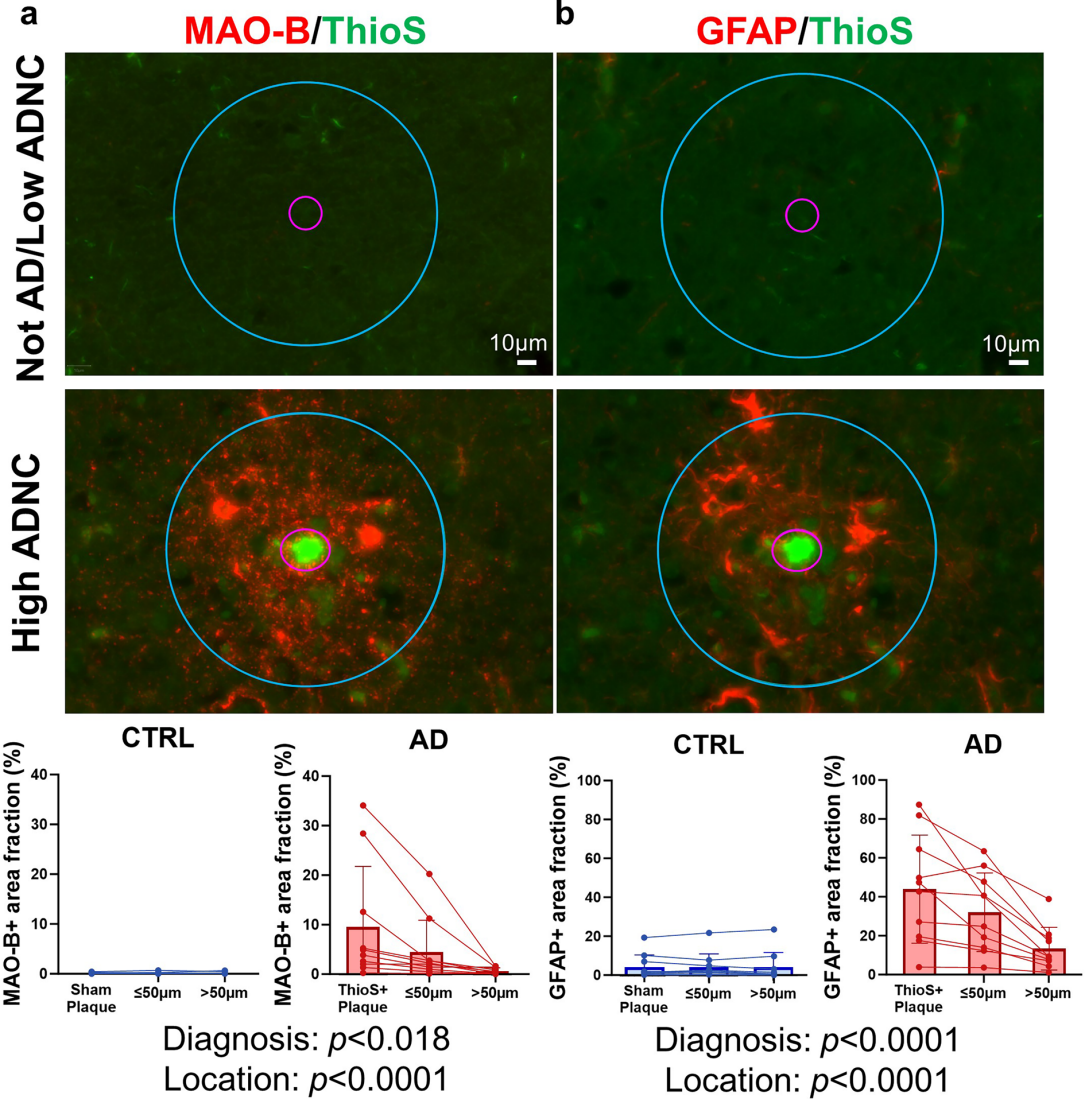
MAO-B expression is increased within and around dense-core Aβ plaques. Plaque-centered analyses in temporal cortex sections from *n* = 10 Not AD/Low ADNC (CTRL) and *n* = 10 High ADNC (AD) doubly immunostained for **a** MAO-B and **b** GFAP (red), and counterstained with Thioflavin-S (ThioS, green) to depict dense-core Aβ plaques. First, 50 ThioS+ plaques per AD donor or 50 sham plaques per CTRL donor were randomly selected and outlined. Next, 50 µm concentric circles were overlaid onto these regions-of-interest to capture the peri-plaque halo. Then, regions-of-interest of similar size were overlaid onto cortical regions distant (> 50 µm) from the nearest ThioS+ or sham plaque. Finally, MAO-B and GFAP area fractions were measured in each of these locations and the effect of location and diagnosis was examined using mixed-effect models (MAO-B ~ location + diagnosis + 1 $\left| { {\text{donor}}\_{\text{ID}}} \right.$). Note that both MAO-B and GFAP were significantly increased in AD versus CTRL donors, particularly within ThioS+ plaques and peri-plaque areas

It should be noted that the Aβ+ area fraction encompasses both diffuse and compact Aβ plaques, and that compact, Thioflavin-S+ (ThioS +) dense-core Aβ plaques are known to trigger more prominent reactive gliosis around them than diffuse deposits [25, 39, 45]. Indeed, MAO-B+ astrocytes appeared to localize around ThioS+ dense-core plaques (Supplemental Fig. S9), whereas diffuse ThioS-Aβ plaques present in some CTRL donors (not AD/low ADNC in Fig. 1b) did not seem to trigger MAO-B upregulation by astrocytes (Supplemental Fig. S10). To unequivocally determine whether MAO-B expression is increased in reactive astrocytes around dense-core plaques, we performed double fluorescent immunohistochemistry for MAO-B and GFAP with ThioS counterstaining in temporal cortex sections from *n* = 10 AD (high ADNC) and *n* = 10 CTRL (not AD/low ADNC) donors. We then quantified MAO-B+ and GFAP+ area fraction within 50 randomly selected ThioS+ plaques, the 50 µm peri-plaque halo, and areas distant (> 50 µm) from the nearest ThioS+ plaque of each AD donor. We did the same in sham plaques randomly placed throughout the temporal cortex of the CTRL donors for comparison. Mixed effect models revealed a significant effect of diagnosis and location on both MAO-B and GFAP immunoreactivity, demonstrating that MAO-B expression is higher in AD versus CTRL, especially in the GFAP+ reactive astrocytes surrounding and penetrating the dense-core Aβ plaques of AD donors (Fig. 6). Indeed, a highly significant (*p* < 0.0001) diagnosis × location interaction (i.e., the closer to ThioS+ plaques from AD donors the higher the MAO-B area fraction versus no change in MAO-B area fraction with distance to sham plaques in CTRL donors) supports this interpretation.

**Fig. 7:**
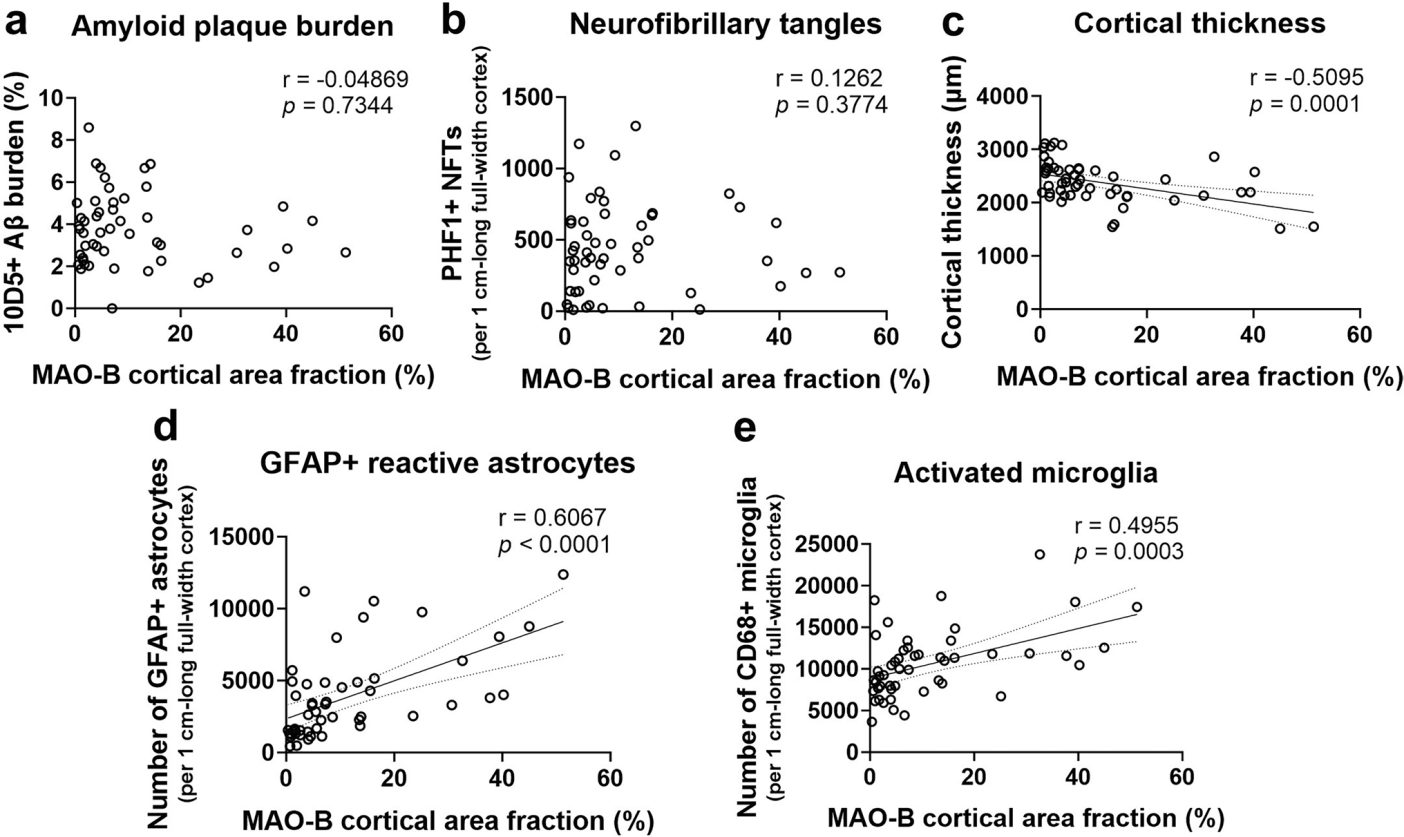
MAO-B expression correlates with local AD neuropathological measures, glial responses, and cortical atrophy in a validation sample. Correlation plots of MAO-B area fraction and **a** Aβ+ area fraction, **b** number of PHF1+ neurofibrillary tangles, **c** cortical thickness, **d** number of GFAP+ reactive astrocytes, and **e** number of CD68+ reactive microglia in the temporal neocortex of a validation sample of donors with intermediate and high ADNC (*n* = 51). Spearman’s rank correlation tests (correlation coefficients and *p* values) are shown in each figure

It should also be noted that the pTau+ area fraction is mainly driven by pTau+ neuropil threads and plaque-associated neuritic dystrophies, whereas neurofibrillary tangles only account for 10% or less of the total pTau+ area fraction [7, 31]. To determine whether the number of pTau+ neurofibrillary tangles indeed correlates with MAO-B expression level, we measured the MAO-B+ area fraction in the temporal cortex of a validation sample of intermediate and high ADNC donors (*n* = 51) for which FFPE sections were available and stereology-based counts of PHF1+ neurofibrillary tangles, GFAP+ reactive astrocytes, and CD68+ microglia as well as Aβ+ area fraction and cortical thickness measures had been obtained for a previous study [44]. Univariate correlations revealed statistically significant positive correlations between MAO-B+ area fraction and the number of GFAP+ reactive astrocytes and CD68+ reactive microglia as well as a statistically significant negative correlation between MAO-B+ area fraction and cortical thickness, but no significant correlation between MAO-B+ area fraction and either Aβ plaque burden or the number of PHF1+ neurofibrillary tangles (Fig. 7 and Table 5). A multivariate regression analysis with MAO-B area fraction as outcome variable, Aβ+ area fraction, number of pTau+ neurofibrillary tangles, GFAP+ reactive astrocytes, and CD68+ reactive microglia, and cortical thickness as independent variables, and adjusted by age at death, confirmed that MAO-B expression level is independently associated with cortical thickness and number of CD68+ reactive microglia and showed a trend (*p* = 0.0546) toward a positive independent association with the number of GFAP+ reactive astrocytes, but no significant association with either total Aβ plaque burden or the number of neurofibrillary tangles (Table 6).

**Table 5:** Univariate correlations of quantitative neuropathological measures obtained in the temporal association neocortex of a validation sample with intermediate and high ADNC burden

	MAO-B+ area fraction	Aβ+ area fraction	pTau+ neurofibrillary tangles	Cortical thickness	GFAP+ reactive astrocytes	CD68+ reactive microglia
MAO-B+ area fraction	1.0					
Aβ+ area fraction	− 0.0487^a^^,^^ns^	1.0				
pTau+ neurofibrillary tangles	0.1262^a,ns^	0.2366^b,ns^	1.0			
Cortical thickness	− 0.5095^a^^,^***	− 0.0824^b,ns^	0.1392^b,ns^	1.0		
GFAP+ reactive astrocytes	0.6067^a^^,^****	− 0.0520^a,ns^	0.3020^a^^,^*	− 0.3584^a^^,^*	1.0	
CD68+ reactive microglia	0.4955^a^^,^***	− 0.0037^a,ns^	0.4703^a^^,^***	− 0.0309^a,^^ns^	0.5140^a^^,^***	1.0

*p < 0.05; **p < 0.01; ***p < 0.001; ****p < 0.0001

^a^Spearman’s rank test

^b^Pearson’s correlation; ns = non-significant

**Table 6:** Multivariable linear regression analysis of MAO-B expression in the temporal association neocortex of the validation sample with intermediate and high ADNC burden

Variables	Estimate	95% CI	*p* value
Age	0.08954	− 0.2992 to 0.4783	0.6446
Aβ+ area fraction	− 0.8351	− 2.753 to 1.083	0.3849
pTau+ neurofibrillary tangles	− 0.004533	− 0.01445 to 0.005384	0.3618
Cortical thickness	− 0.01196	− 0.01984 to − 0.004074	0.0038
GFAP+ reactive astrocytes	0.001121	− 2.296e-005 to 0.002265	0.0546
CD68+ reactive microglia	0.001369	0.0005345 to 0.002203	0.0019

### MAO-B expression is not impacted by the *MAOBrs1799836* SNP genotype

**Fig. 8:**
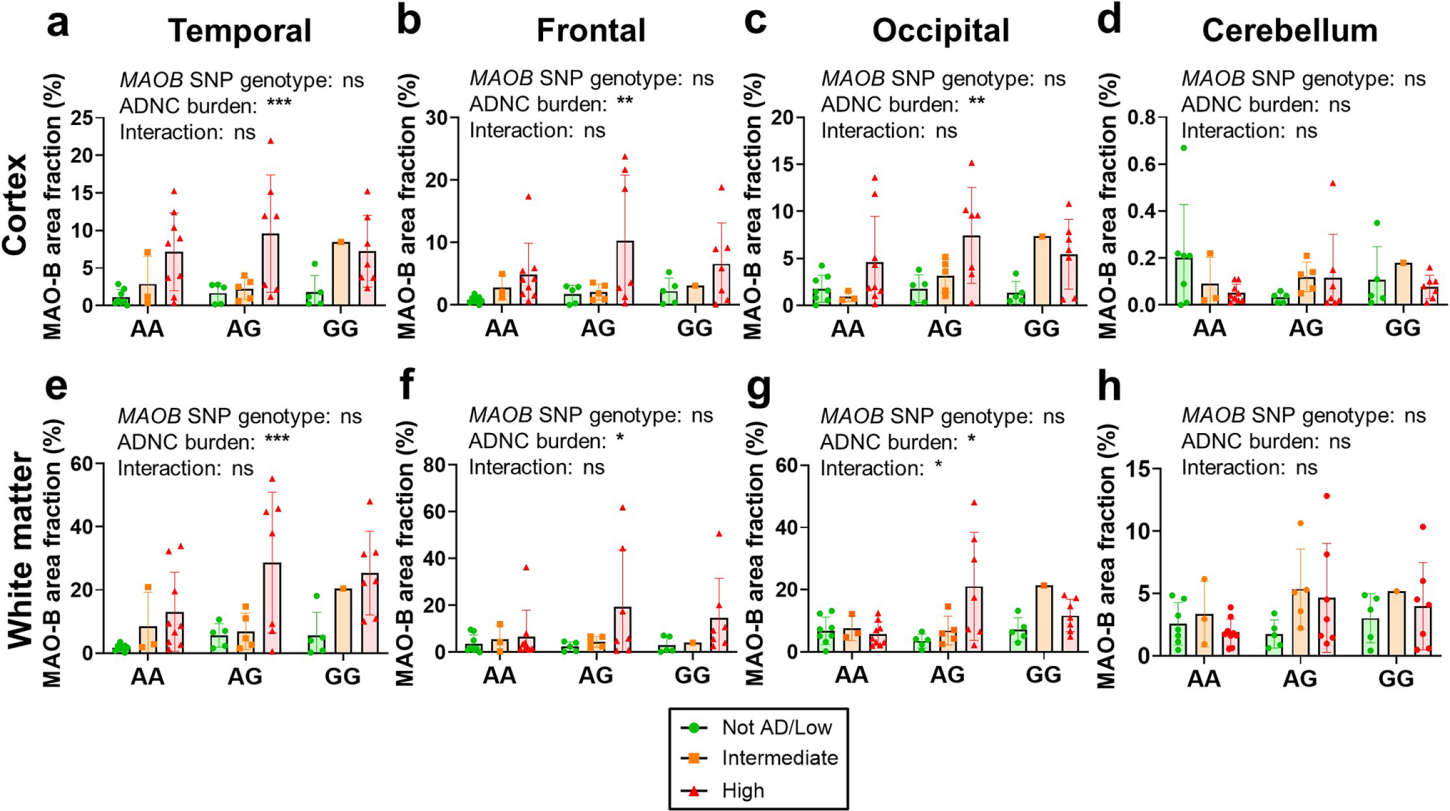
*MAOB rs1799836* SNP genotype does not impact MAO-B expression level across brain regions. Two-way ANOVA with ADNC burden and *MAOB rs1799836* SNP genotype as cofactors demonstrated no effect of *MAOB rs1799836* SNP genotype on MAO-B expression level in **a** temporal cortex, **e** temporal white matter, **b** frontal cortex, **f** frontal white matter, **c** occipital cortex, **g** occipital white matter, **d** cerebellar cortex, and **h** cerebellar white matter (ns = non-significant; *p < 0.05, **p < 0.01, ***p < 0.001, two-way ANOVA)

We also investigated the possible impact of the *MAOB rs1799836* SNP genotype on MAO-B expression level, since its G (minor) allele has been associated with lower MAO-B enzymatic activity [3], which could potentially affect radiotracer binding. Of the 50 donors in whom this SNP genotyping could be assayed, 20 were AA, 17 AG, and 13 GG. MAO-B+ area fraction increased with increasing ADNC burden in all brain regions regardless of the *MAOB rs1799836* SNP genotype, indicating no effect of this SNP on MAO-B expression level (Fig. 8).

### MAO-B expression is also increased in some ADRD

**Fig. 9:**
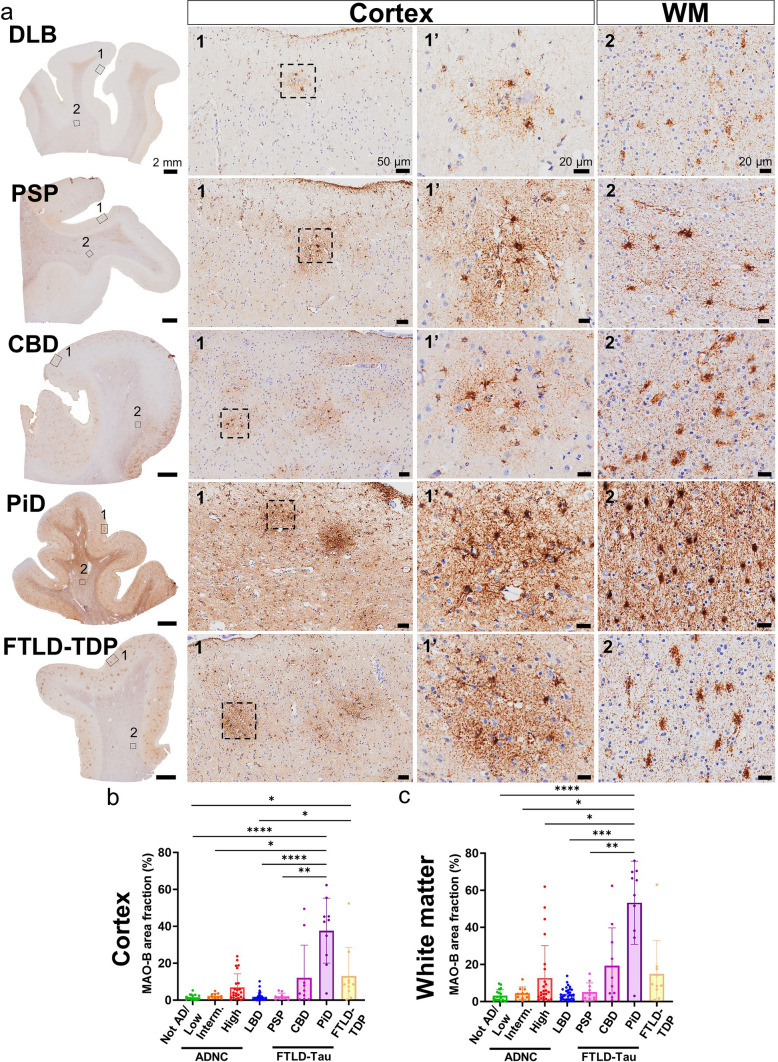
MAO-B is upregulated by cortical and white matter astrocytes in some ADRD. Photomicrographs of MAO-B immunohistochemistry with the peroxidase-DAB method in the frontal association cortex (BA8/9) of representative study donors with various ADRD. Note that MAO-B is mainly expressed by astrocytes also in ADRD. Scale bars: whole-slide images, 2 mm; insets #1, 50 μm; and insets #1′ and #2, 20 μm. **b**, **c** Comparative analysis of MAO-B expression across AD/ADRC revealed that MAO-B immunoreactive area fraction is highest in donors with PiD, followed by CBD, FTLD-TDP, and AD (High ADNC), and lowest in CTRL (Not AD/Low ADNC) donors, followed by intermediate ADNC, LBD, and PSP donors in both (b) cortex and (c) white matter. Note that MAO-B area fraction is higher in white matter than cortex across AD/ADRD (ns = non-significant; *p < 0.05, **p < 0.01, ****p < 0.0001, Kruskal–Wallis ANOVA with Dunn's post-test; LBD = Lewy body disease, PiD = Pick’s disease, PSP = progressive supranuclear palsy; CBD = corticobasal degeneration, FTLD-Tau = frontotemporal lobar degeneration-tau, FTLD-TDP = frontotemporal lobar degeneration—Tar DNA-binding protein of 43 kDa)

Lastly, we asked if this MAO-B upregulation by reactive astrocytes is specific of AD or also occurs in ADRD. To address this question, we performed quantitative immunohistochemistry with the peroxidase-DAB method followed by the same analytical pipeline in FFPE sections from the frontal association cortex (BA8/9) of *n* = 30 LBD and *n* = 10 of each PiD, PSP, CBD, and FTLD-TDP donors. We chose this brain region because it is known to be significantly affected in all these ADRD [2, 8]. We found that, in these conditions, astrocytes are also the main source of MAO-B expression in both cortex and white matter (Fig. 9a). Surprisingly, quantitative analyses of MAO-B-immunoreactive area fraction revealed that CBD, PiD, and FTLD-TDP donors exhibit an even higher MAO-B expression than donors with high ADNC burden and much higher than CTRL donors (Not AD/Low ADNC burden). By contrast, MAO-B area fraction in LBD and PSP donors did not differ from that of CTRL donors (Fig. 9b, c).

## Discussion

Our findings can be summarized as follows: (1) in CTRL brains, MAO-B is primarily expressed by subpial and perivascular astrocytes in the cortex and by perivascular white matter astrocytes; (2) MAO-B expression level is significantly increased in multiple brain regions in AD versus CTRL brains and in both cortex and white matter, but not in cerebellum; (3) MAO-B is upregulated by reactive astrocytes within and around dense-core, ThioS+ Aβ plaques in AD brains; (4) MAO-B expression level independently correlates with local measures of reactive astrocytes and microglia, and cortical atrophy; (5) the *MAOB rs1799836* SNP genotype does not significantly impact MAO-B expression level; and (6) MAO-B expression is also upregulated by frontal cortical and white matter astrocytes in PiD, CBD, and FTLD-TDP, but not in LBD or PSP.

These findings have several important implications for PET imaging of reactive astrogliosis. First, the predominant expression of MAO-B by astrocytes relative to other cell types across cortical brain areas and its upregulation by reactive astrocytes in the vicinity of dense-core Aβ plaques support the validity of MAO-B as a target for PET radiotracers of AD-associated reactive astrogliosis. Discrepancies between our study and prior works describing an upregulation of MAO-B in cortical and hippocampal pyramidal neurons might be explained by differences in MAO-B antibody used and/or epitope targeted, immunohistochemistry method (automated vs. manual), and tissue fixation [42]. Second, the low expression of MAO-B in cerebellum, especially in cerebellar cortex, supports this choice as reference region for MAO-B PET radiotracer uptake quantification [40]. Third, the higher MAO-B expression in white versus gray matter in all cortical areas and diagnoses analyzed underscores the need for co-registration of PET images with T1-weighted-based MRI sequences to differentiate white matter from cortex and specifically assess cortical reactive astrogliosis [40], and argues against using white matter as the reference region to measure cortical radiotracer uptake [12, 40, 46–48]. Fourth, overall the *MAOB rs1799836* SNP genotype did not impact MAO-B expression level, which argues against the need for genotyping this SNP to interpret PET results. Last, MAO-B-based PET imaging could be useful to depict reactive astrogliosis not only in AD but also in some ADRD.

In addition, our findings of MAO-B upregulation by AD reactive astrocytes have important pathophysiological implications. On one hand, MAO-B enzymatic activity generates hydrogen peroxide—one of the main reactive oxygen species (ROS)—suggesting that reactive astrocytes in the AD brain and some ADRD exhibit a gain of toxic function that could potentially accelerate the rate of neurodegeneration [13]. Of note, it has been reported that Aβ plaques are a reservoir of ROS and that this oxidative stress drives, at least partly, the dystrophic neurites that decorate neuritic Aβ plaques [17, 18]. Thus, mitochondria from reactive astrocytes may be one of the main cellular sources of ROS in the AD brain. On the other hand, MAO-B enzymatic activity can ultimately lead to GABA synthesis and release by astrocytes [52] and reactive astrocytes have been implicated in cognitive deficits in mouse models of AD via GABA-mediated tonic inhibition of neuronal circuits [26, 49].

Moreover, our multivariable regression analysis revealed that MAO-B expression level is independently associated with local measures of reactive gliosis and cortical atrophy, raising the possibility that MAO-B-based PET imaging could be helpful to predict the rate of AD progression. Notably, plasma GFAP levels have been recently shown to correlate positively with ^18^F-SMBT-1 uptake [10]—but, surprisingly, negatively with ^11^C-DED uptake [12]—in several brain regions. The lack of significant association between MAO-B expression level and Aβ plaque burden in our multivariable regression analysis deserves further discussion. Our Aβ+ area fraction encompasses both diffuse (usually non-neuritic and not triggering an astrocyte response) and compact plaques (also known as dense-core and fibrillar, which are usually neuritic and surrounded by reactive astrocytes). Our pTau+ area fraction is mostly reflecting the pTau+ dystrophic neurites of the neuritic Aβ plaques and neuropil threads rather than the number of neurofibrillary tangles [7, 31]. MAO-B immunoreactivity correlated positively with pTau+ area fraction (Table 4) but not with the number of pTau+ neurofibrillary tangles (Table 6), suggesting that neuritic plaques, rather than tangles, are the main driver of MAO-B upregulation by astrocytes in AD. Our spatial quantitative analyses centered around ThioS+ dense-core plaques (usually laden with pTau+ dystrophic neurites) further support this conclusion by demonstrating that reactive astrocytes around this subset of plaques are highly immunoreactive for MAO-B. Aβ PET radiotracers preferentially detect dense-core (fibrillar) Aβ plaques [24, 30] and, therefore, we may expect a positive correlation between Aβ and MAO-B PET radiotracer uptakes, as shown by Chatterjee et al. [10].

The analysis of MAO-B expression levels in the frontal lobe of ADRD donors indicate that MAO-B upregulation by reactive astrocytes is not exclusively associated with the dense-core neuritic plaques of AD. The sharp differences across FTLD-Tau subtypes are striking and reinforce the idea that reactive astrogliosis is a context-dependent phenomenon [14, 16]. The utility of MAO-B-based PET imaging remains to be thoroughly examined in ADRD; Chiotis et al. have recently found increased ^11^C-DED binding in frontotemporal areas in five patients with semantic variant primary progressive aphasia (svPPA, neuropathologically typically corresponding to FTLD-TDP) and five with behavioral variant frontotemporal dementia (neuropathologically usually corresponding to either FTLD-TDP or FTLD-Tau) [11], whereas Ballweg et al. have reported low binding of ^18^F-DED in the cortex of two patients with Parkinson disease (PD) but very high in two patients with multiple system atrophy (MSA), especially in the parkinsonian type [4]. To inform future MAO-B-based PET imaging studies in ADRD, it will be important to investigate possible correlations between MAO-B area fraction and both the local burden of protein aggregates (α-synuclein, pTau, and pTDP-43 in LBD, FTLD-Tau, and FTLD-TDP, respectively) and the severity of cortical atrophy in postmortem brains of donors with these primary neuropathological diagnoses.

Lastly, our findings may have some therapeutic implications. If MAO-B upregulation by reactive astrocytes is confirmed to promote neurodegeneration as suggested by preclinical studies [13, 26, 49, 52], MAO-B inhibition could have therapeutic value to slow down AD/ADRD progression. However, while MAO-B inhibitors selegiline (L-deprenyl) and rasagiline are approved for the symptomatic treatment of PD, clinical trials with MAO-B inhibitors have shown no clear benefit to slow down AD-related cognitive decline [6, 33].

Some limitations of our study should also be acknowledged. Neuropathological studies are inherently cross-sectional and could be confounded by agonal perimortem factors. MAO-B-immunoreactive area fraction in postmortem FFPE brain tissue sections may be an imperfect proxy for PET imaging of reactive astrogliosis, as it may not correlate well with PET radiotracer uptake in vivo, which is affected not only by the expression levels of the radiotracer target but also by radiotracer-specific pharmacokinetic and pharmacodynamic factors [40]. Nuclear emulsion autoradiography combined with immunohistochemistry in cryostat sections could enable the identification of the specific cell type(s) that the actual MAO-B radiotracers bind to [1]. While we did not observe an association between MAO-B expression level and the *MAOB rs1799836* genotype, the minor allele has been previously correlated with a reduced brain MAO-B enzymatic activity [3], which could lead to lower radiotracer binding; thus, we cannot rule out a scenario similar to that of TSPO-based PET imaging, in which the *TSPO* *rs6971* SNP genotype does not impact brain TSPO expression level [19, 20] but may affect the binding affinity of some TSPO radiotracers [22, 28, 35, 51].

In summary, we conclude that MAO-B-based PET imaging is a promising biomarker of reactive astrogliosis and may also be a valuable biomarker of AD progression. Larger and longer longitudinal multimodal imaging and fluid biomarker studies are needed to correlate A/T/N and other glia-specific biomarkers with MAO-B PET radiotracer uptake and confirm the associations we found in postmortem brains [41, 46].

### Supplementary Information

Below is the link to the electronic supplementary material.

Supplementary file1 (PDF 17082 kb)
