# Supplementary material for: Characterization of monoamine oxidase-B (MAO-B) as a biomarker of reactive astrogliosis in Alzheimer’s disease and related dementias
Source: Acta Neuropathol. 2024 Apr 3;147(1):66. doi: 10.1007/s00401-024-02712-2 (PMC10991006; doi:10.1007/s00401-024-02712-2)
Supplement: Supplementary file 1 — Supplementary file1 (PDF 17082 kb) [file 401_2024_2712_MOESM1_ESM.pdf]

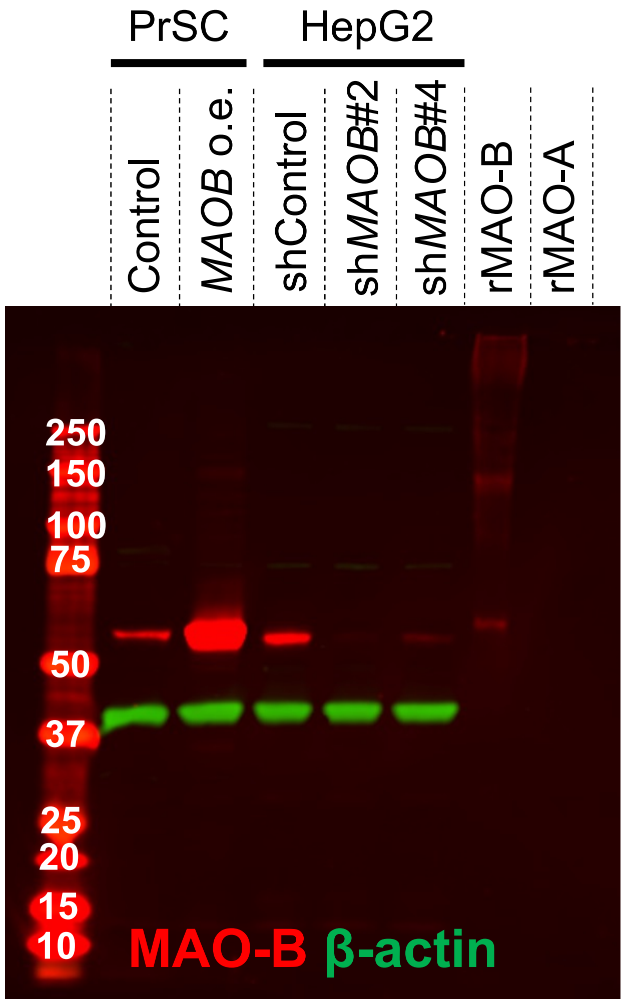

PrSC
HepG2
Control
MAOB o.e.
shControl
shMAOB#2
shMAOB#4
rMAO-B
rMAO-A
250
150
100
75
50
37
25
20
15
10
MAO-B β-actin

Supplemental Figure 1. Validation of anti-MAO-B antibody used for immunohistochemical studies.

Western blot on lysates of human prostate stromal carcinoma (PrSC) cells stably transfected with control or *MAOB* plasmid, lysates of human hepatocarcinoma (HepG2) cells stably transfected with control shRNA or two different anti-*MAOB* shRNAs (#2 and #4), and recombinant proteins MAO-B and MAO-A. Note the single band of ~59 kDa that increases in MAO-B overexpressing cells and virtually disappears in cells transfected with specific shRNAs. Note also the lack of cross-reactivity with recombinant MAO-A. The higher molecular weight of recombinant MAO-B (~62 kDa) is likely due to the 6xHis-tag used for its purification.

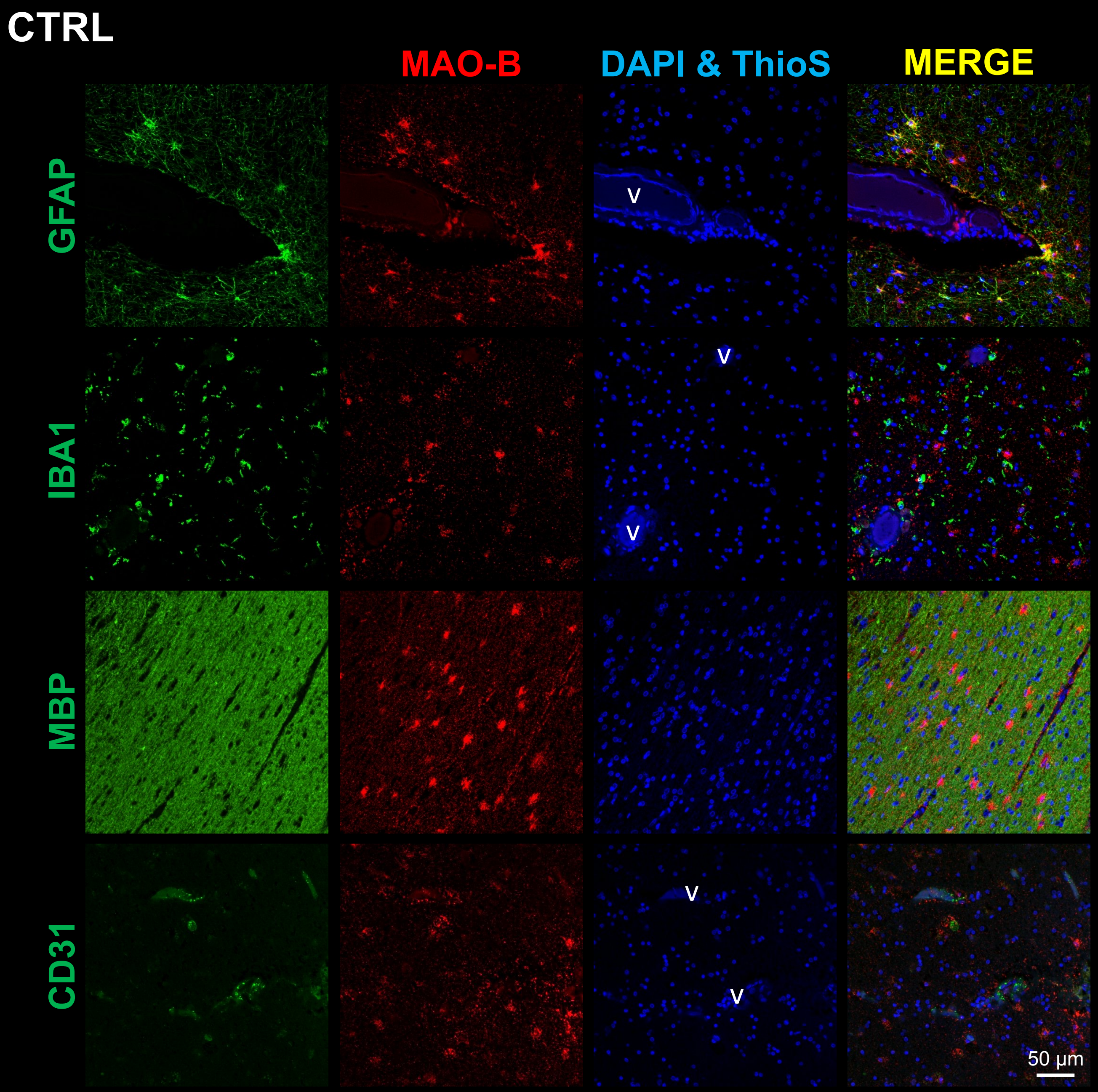
CTRL
MAO-B
DAPI & ThioS
MERGE
GFAP
IBA1
MBP
CD31
V
V
V
V
V
50 µm

Supplemental Figure 2. Astrocytes are the main cell type expressing MAO-B in control white matter.

Double fluorescent immunohistochemistry for MAO-B and cell-type specific markers of either astrocytes (GFAP), microglia (IBA1), oligodendrocytes (MBP), or endothelial cells (CD31) in the temporal white matter of representative CTRL donors. Confocal microscopy images reveal that MAO-B co-localizes predominantly with perivascular astrocytes in the CTRL white matter, but not with the other cell types. Scale bar: 50 μm.

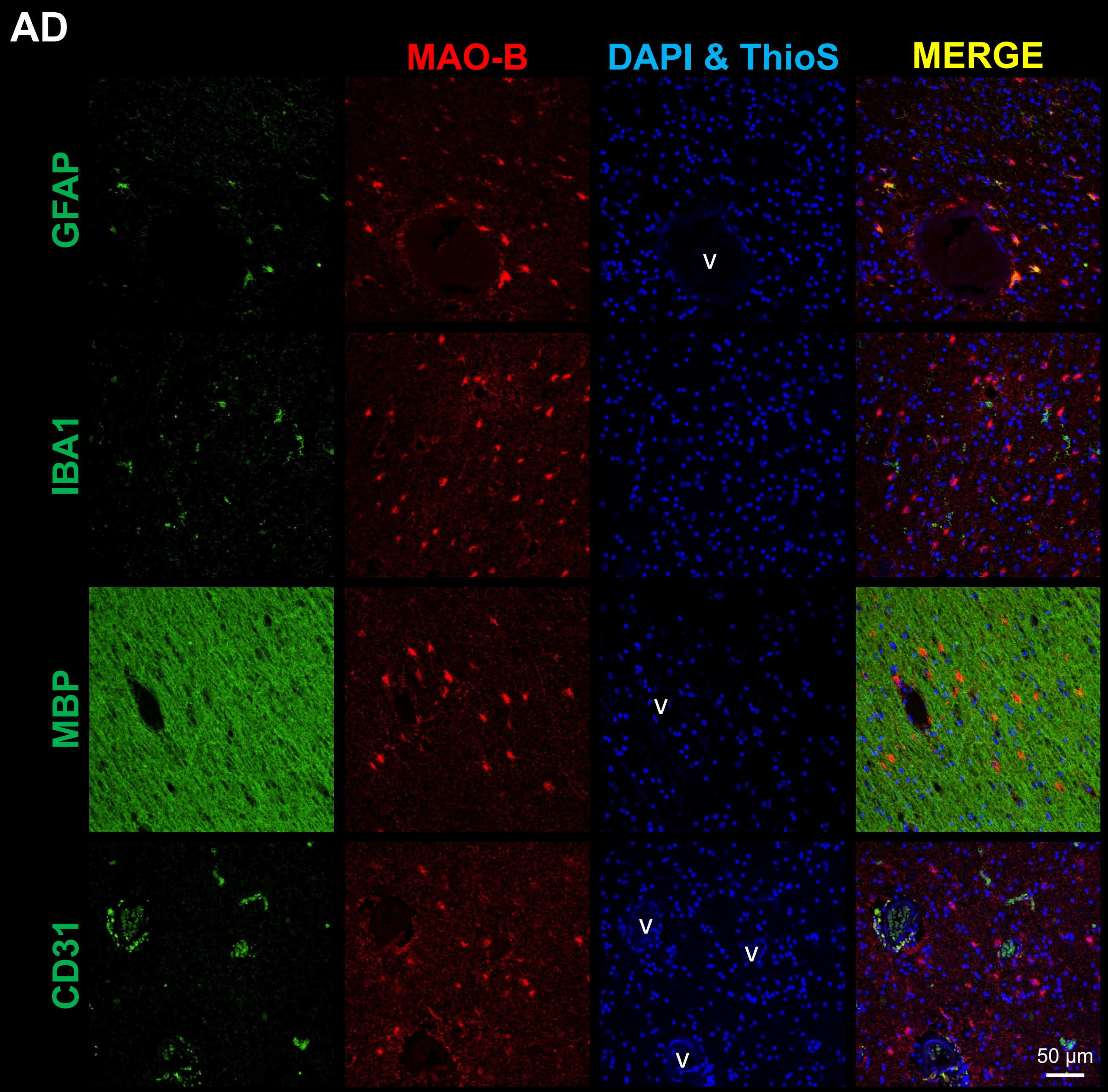
AD
MAO-B
DAPI & ThioS
MERGE
GFAP
IBA1
MBP
CD31
V
V
V
V
V
50 µm

Supplemental Figure 3. Astrocytes are the main cell type expressing MAO-B in the AD white matter.

Double fluorescent immunohistochemistry for MAO-B and cell-type specific markers of either astrocytes (GFAP), microglia (IBA1), oligodendrocytes (MBP), or endothelial cells (CD31) in the temporal white matter of representative AD donors. Confocal microscopy images show that MAO-B co-localizes with GFAP+ astrocytes in the AD white matter, but not with the other cell types. Scale bar: 50 μm.

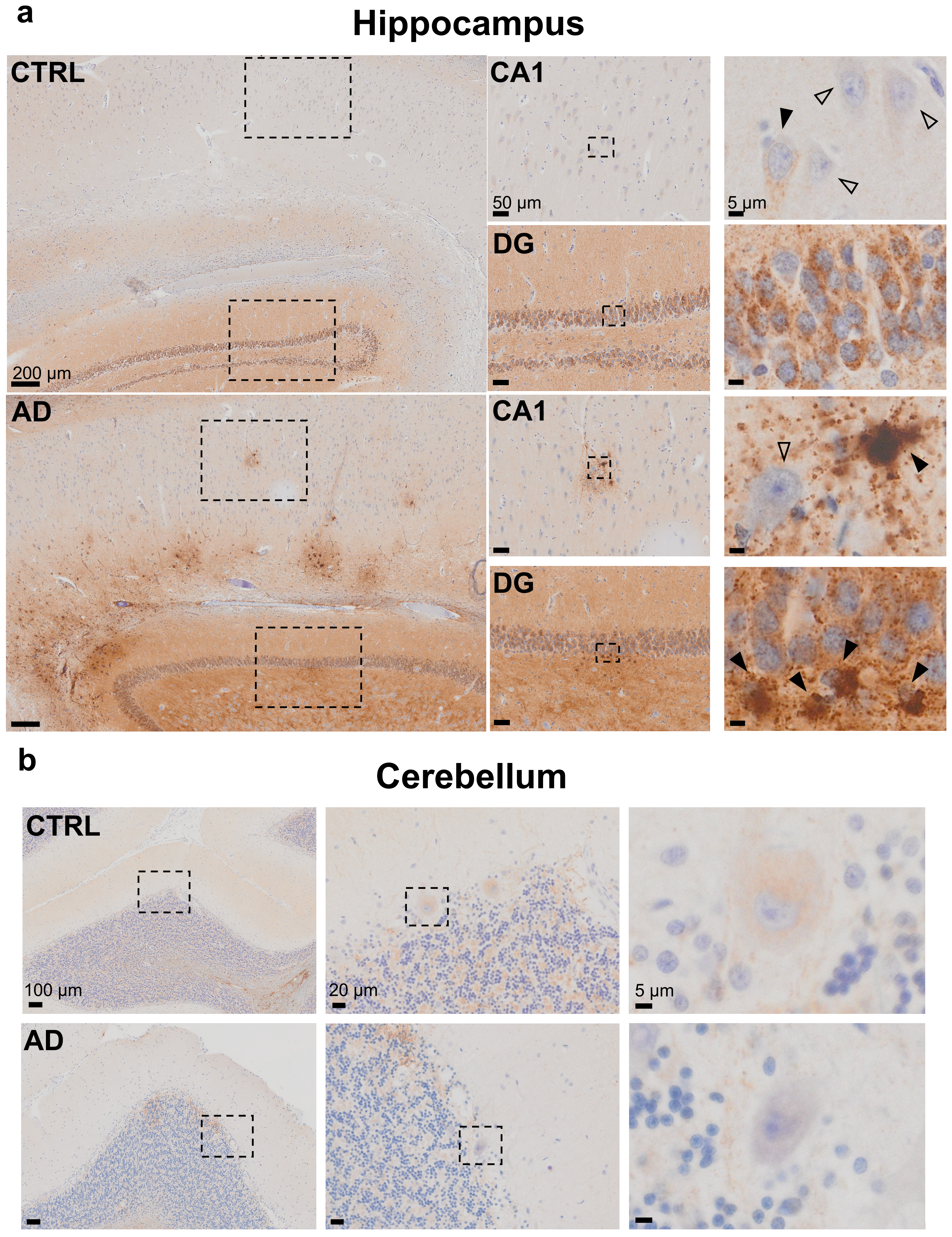
a
Hippocampus
CTRL
CA1
50 μm
5 μm
DG
200 μm
AD
CA1
DG
b
Cerebellum
CTRL
100 μm
20 μm
5 μm
AD

Supplemental Figure 4. Pattern of MAO-B expression in the hippocampus and cerebellum. Representative photomicrographs of MAO-B immunohistochemistry with the peroxidase-DAB method in the hippocampus (a) and cerebellum (b) from a representative CTRL and AD donors. (a) Rare pyramidal neurons in the CA1 region of CTRL and AD donors were mildly positive for MAO-B, but its expression was much higher in nearby reactive astrocytes. By contrast, granular cells from the dentate gyrus do exhibit significant MAO-B expression in both CTRL and AD donors. Note also the high expression of MAO-B in hilar astrocytes from the AD donor. (b) Little or no expression of MAO-B in cerebellar Purkinje cells. Scale bars: low power — hippocampus 200 μm and cerebellum 100 μm; mid power — hippocampus 50 μm and cerebellum 20 μm; high power — hippocampus and cerebellum 5 μm.

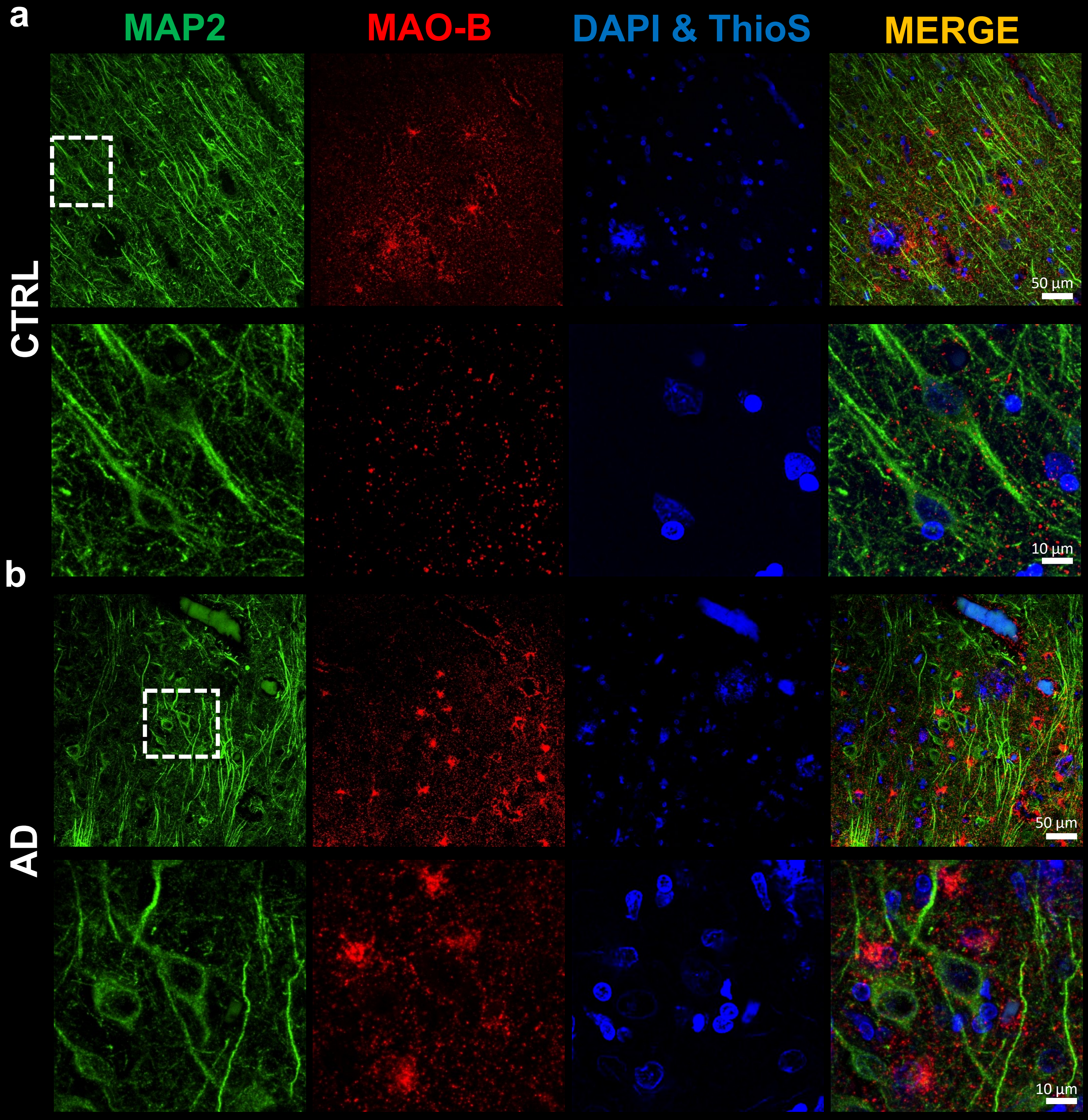
a
MAP2
MAO-B
DAPI & ThioS
MERGE
CTRL
50 µm
10 µm
b
AD
50 µm
10 µm

Supplemental Figure 5. Comparison of astrocytic vs. neuronal MAO-B expression. Confocal microscopy photomicrographs of layers II-III of the temporal association neocortex from a representative CTRL (a) and AD (b) donors showing little if any MAO-B expression (red) in MAP2+ neurons (green). Conversely, note the high expression of MAO-B in cells with astrocyte morphology, particularly in the AD donor. Similar findings apply to deeper layers of the cortex. Scale bars: 50 μm, insets 10 μm.

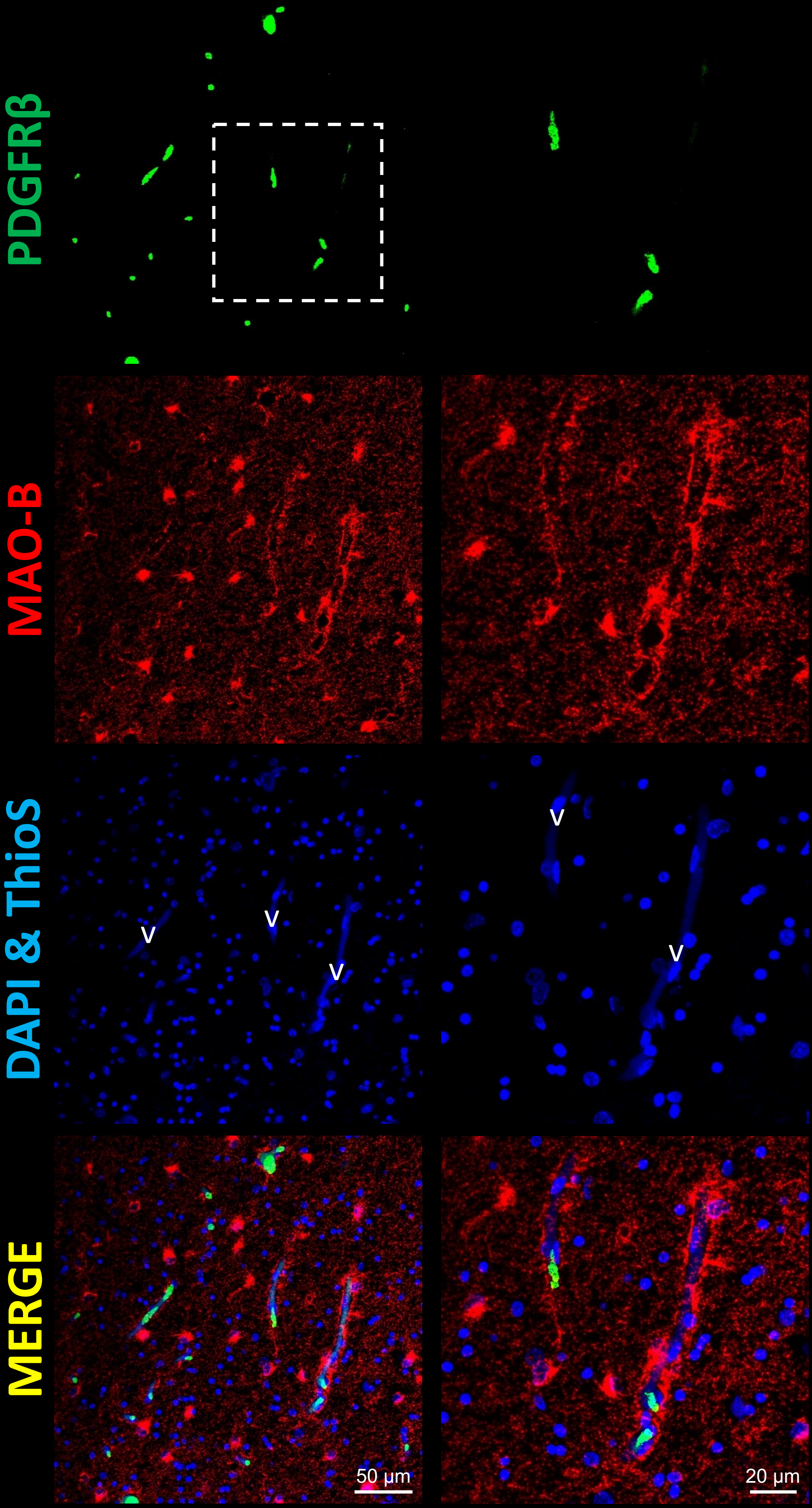
PDGFRβ
MAO-B
DAPI & ThioS
V
V
V
V
V
MERGE
50 μm
20 μm

Supplemental Figure 6. Pericytes do not express MAO-B.

Double fluorescent immunohistochemistry for MAO-B and the pericyte marker PDGFRβ in the temporal association cortex of an AD donor. Confocal microscopy images show that MAO-B is expressed by peri-capillary astrocytes but does not co-localize with pericytes. *Left*, low power field (scale bar: 50 μm); *right*, higher power inset (scale bar: 20 μm) corresponding to the dashed box in the left. V = vessel.

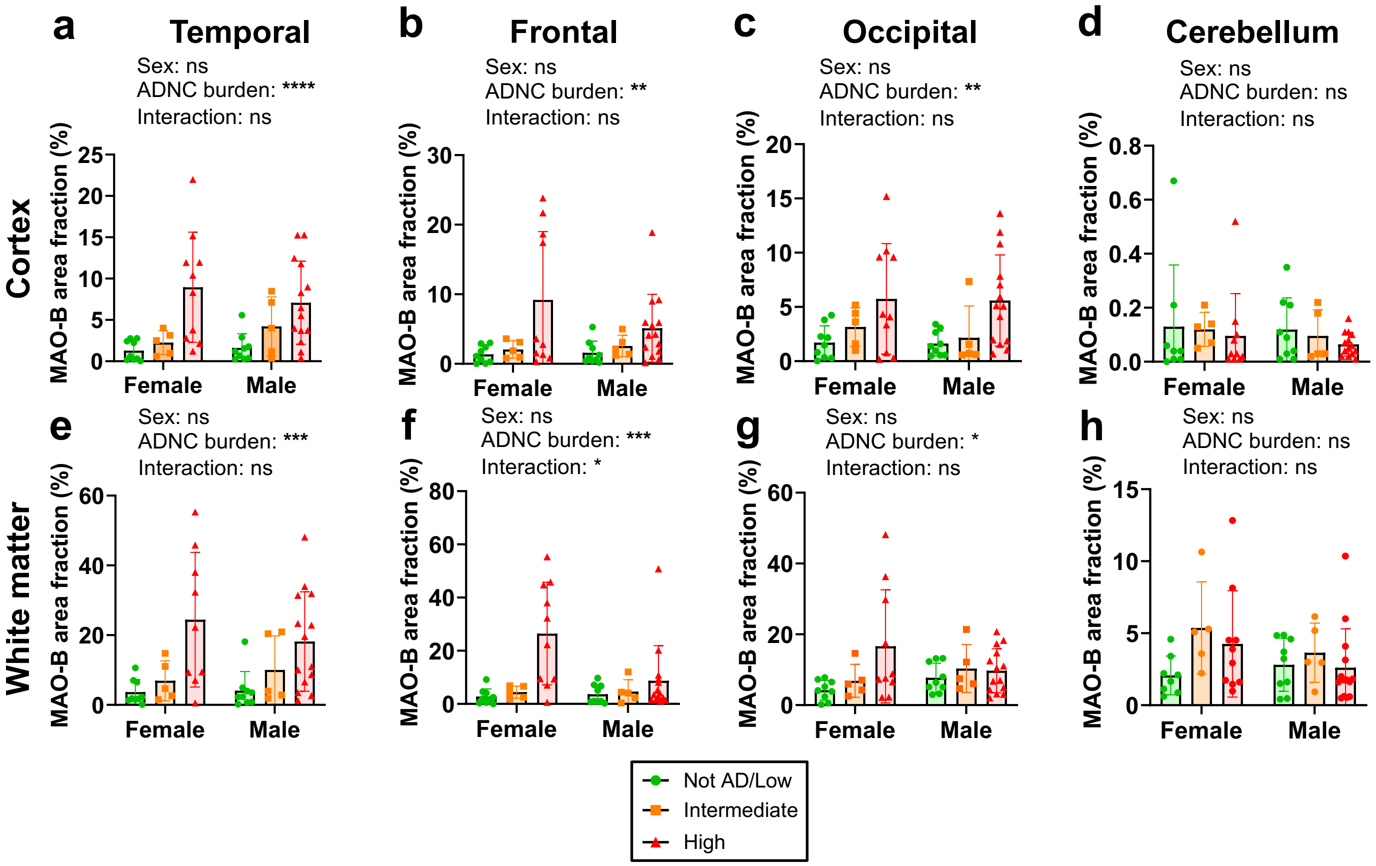

a
Temporal
Sex: ns
ADNC burden: ****
Interaction: ns
b
Frontal
Sex: ns
ADNC burden: **
Interaction: ns
c
Occipital
Sex: ns
ADNC burden: **
Interaction: ns
d
Cerebellum
Sex: ns
ADNC burden: ns
Interaction: ns
Cortex
MAO-B area fraction (%)
Female
Male
e
Sex: ns
ADNC burden: ***
Interaction: ns
f
Sex: ns
ADNC burden: ***
Interaction: *
g
Sex: ns
ADNC burden: *
Interaction: ns
h
Sex: ns
ADNC burden: ns
Interaction: ns
White matter
Not AD/Low
Intermediate
High

Supplemental Figure 7. Sex does not impact MAO-B expression level across brain regions.

Two-way ANOVA with ADNC burden and sex as cofactors demonstrated no effect of sex on MAO-B expression level in (a) temporal cortex, (e) temporal white matter, (b) frontal cortex, (f) frontal white matter, (c) occipital cortex, (d) cerebellar cortex, and (h) cerebellar white matter. Only (g) occipital white matter exhibited a significant ADNC burden × sex interaction (i.e., higher MAO-B+ area fraction in females), the relevance of which is uncertain (ns = non-significant; * $p < 0.05$, ** $p < 0.01$, *** $p < 0.001$, **** $p < 0.0001$, two-way ANOVA).

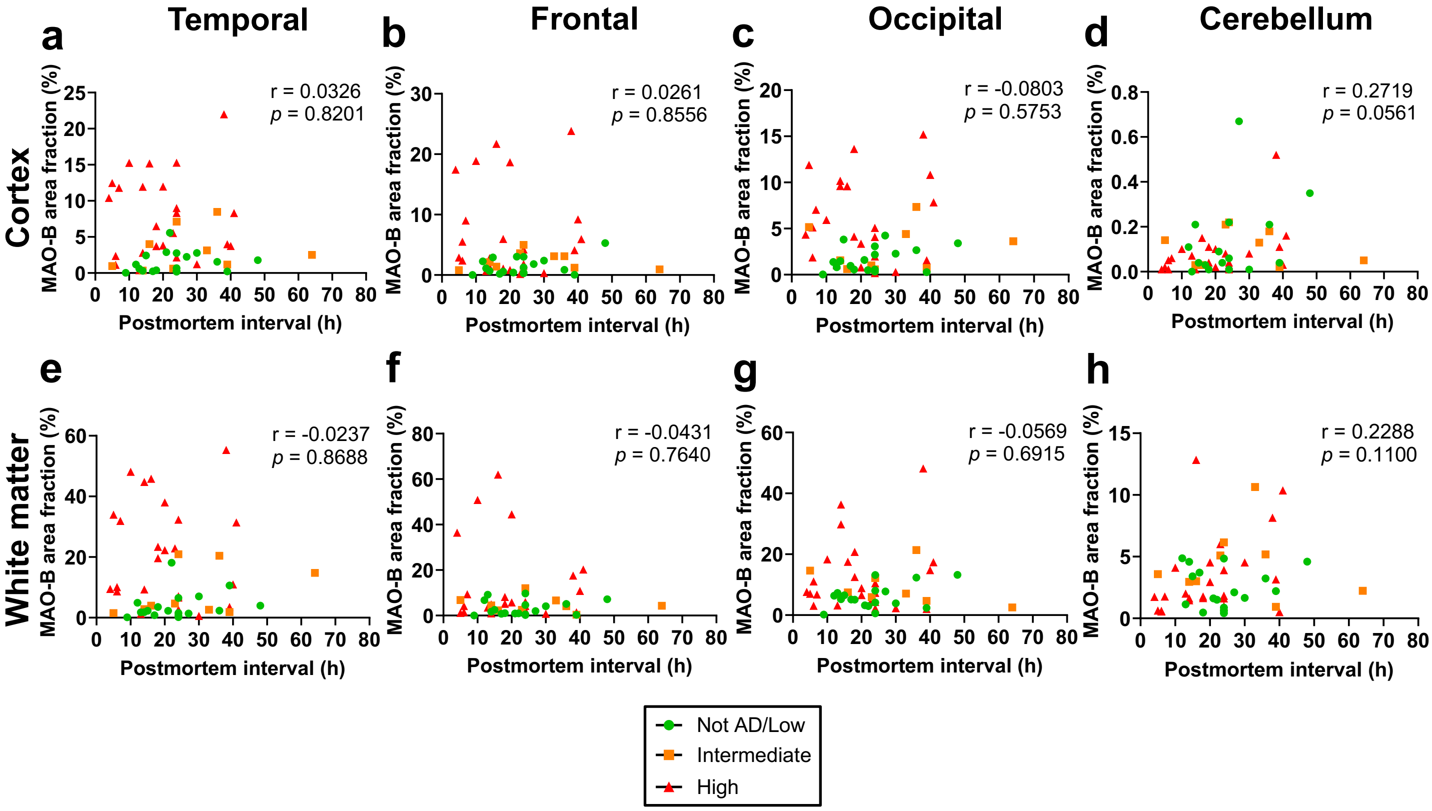

Temporal
Frontal
Occipital
Cerebellum
Cortex
White matter
a
b
c
d
e
f
g
h
r = 0.0326
p = 0.8201
r = 0.0261
p = 0.8556
r = -0.0803
p = 0.5753
r = 0.2719
p = 0.0561
r = -0.0237
p = 0.8688
r = -0.0431
p = 0.7640
r = -0.0569
p = 0.6915
r = 0.2288
p = 0.1100
MAO-B area fraction (%)
Postmortem interval (h)
Not AD/Low
Intermediate
High

Supplemental Figure 8. Postmortem interval does not correlate with MAO-B expression level across brain regions.

Correlation plots of MAO-B area fraction and postmortem interval (in hours) in (a) temporal cortex, (e) temporal white matter, (b) frontal cortex, (f) frontal white matter, (c) occipital cortex, (d) cerebellar cortex, (g) occipital white matter, and (h) cerebellar white matter. Spearman's rank correlation tests (correlation coefficients and *p* values) are shown in each figure.

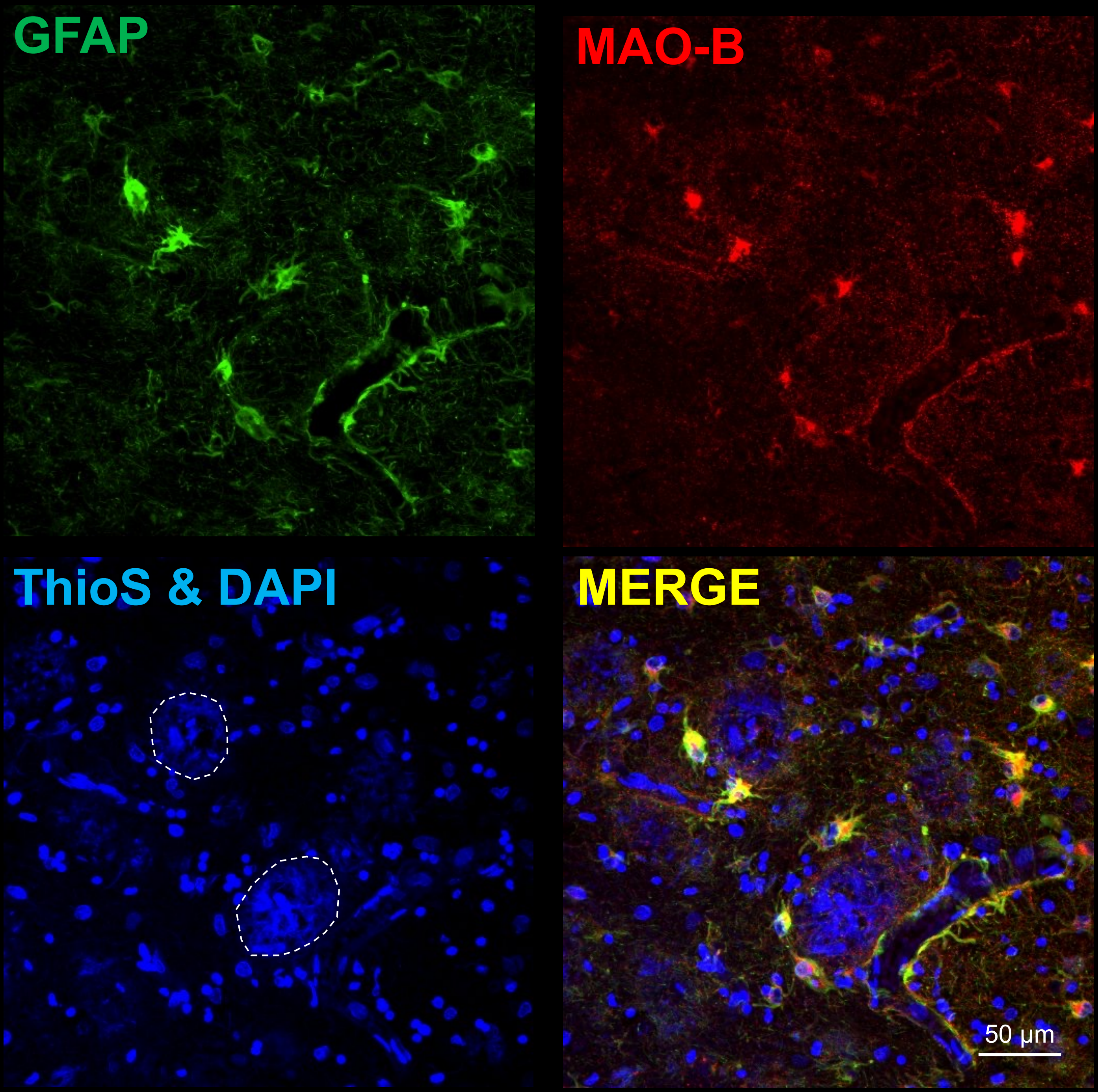
GFAP
MAO-B
ThioS & DAPI
MERGE
50 µm

Supplemental Figure 9. MAO-B is expressed in reactive astrocytes surrounding dense-core Aβ plaques.

Double fluorescent immunohistochemistry for MAO-B and GFAP with Thioflavin-S counterstaining in the temporal association cortex of a representative AD donor. Confocal microscopy images show that MAO-B is upregulated by GFAP+ astrocytes surrounding two dense-core ThioS+ Aβ plaques. Note also some perivascular astrocytes immunoreactive for both GFAP and MAO-B in the right. Scale bar: 50 μm.

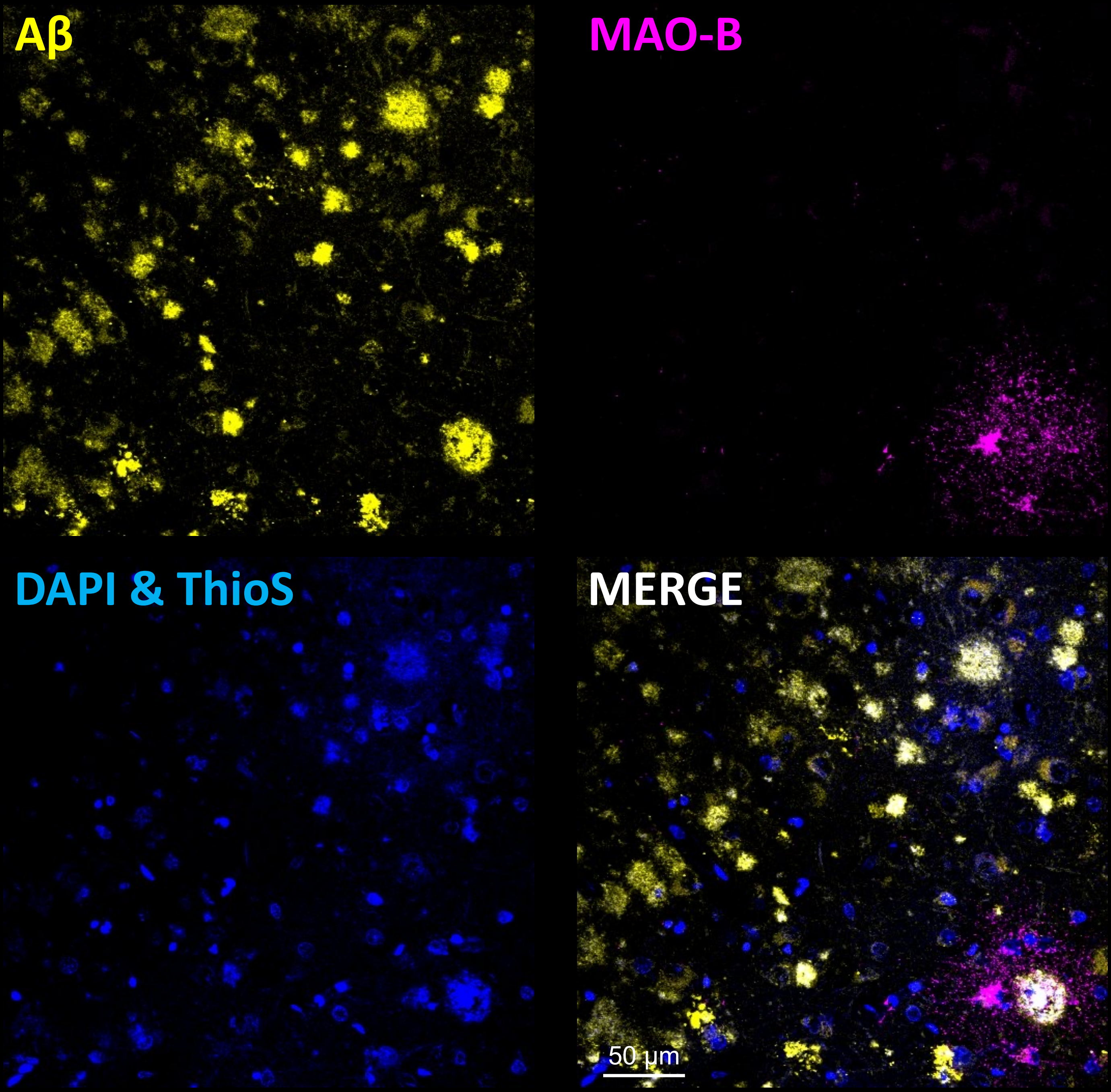
Aβ
MAO-B
DAPI & ThioS
MERGE
50 µm

Supplemental Figure 10. Diffuse Aβ plaques are not associated with MAO-B+ astrocytes.

Double fluorescent immunohistochemistry for MAO-B and Aβ with Thioflavin-S counterstaining in the frontal cortex of a CTRL donor with abundant diffuse Aβ plaques. Confocal microscopy images show MAO-B+ astrocytes near a Thioflavin-S+ dense-core plaque whereas diffuse Aβ deposits are devoid of MAO-B+ reactive astrocytes. Scale bar: 50 μm.
